# Transcriptome driven discovery of novel candidate genes for human neurological disorders in the telomer-to-telomer genome assembly era

**DOI:** 10.1186/s40246-023-00543-y

**Published:** 2023-10-23

**Authors:** Clemens Falker-Gieske

**Affiliations:** Division of Functional Breeding, Department of Animal Sciences, Georg-August-Universität Göttingen, Burckhardtweg 2, 37077 Göttingen, Germany

**Keywords:** Telomer-to-telomer, Human genome, Neurological disorders, Transcriptomics

## Abstract

**Background:**

With the first complete draft of a human genome, the Telomere-to-Telomere Consortium unlocked previously concealed genomic regions for genetic analyses. These regions harbour nearly 2000 potential novel genes with unknown function. In order to uncover candidate genes associated with human neurological pathologies, a comparative transcriptome study using the T2T-CHM13 and the GRCh38 genome assemblies was conducted on previously published datasets for eight distinct human neurological disorders.

**Results:**

The analysis of differential expression in RNA sequencing data led to the identification of 336 novel candidate genes linked to human neurological disorders. Additionally, it was revealed that, on average, 3.6% of the differentially expressed genes detected with the GRCh38 assembly may represent potential false positives. Among the noteworthy findings, two novel genes were discovered, one encoding a pore-structured protein and the other a highly ordered β-strand-rich protein. These genes exhibited upregulation in multiple epilepsy datasets and hold promise as candidate genes potentially modulating the progression of the disease. Furthermore, an analysis of RNA derived from white matter lesions in multiple sclerosis patients indicated significant upregulation of 26 rRNA encoding genes. Additionally, putative pathology related genes were identified for Alzheimer’s disease, amyotrophic lateral sclerosis, glioblastoma, glioma, and conditions resulting from the m.3242 A > G mtDNA mutation.

**Conclusion:**

The results presented here underline the potential of the T2T-CHM13 assembly in facilitating the discovery of candidate genes from transcriptome data in the context of human disorders. Moreover, the results demonstrate the value of remapping sequencing data to a superior genome assembly. Numerous potential pathology related genes, either as causative factors or related elements, have been unveiled, warranting further experimental validation.

**Supplementary Information:**

The online version contains supplementary material available at 10.1186/s40246-023-00543-y.

## Background

With the publication of the first complete sequence of a human genome by the Telomere-to-Telomere (T2T) Consortium [1], a number of shortcomings in the previous gold standard reference assembly, GRCh38 [2], were addressed. The GRCh38 genome assembly contains gaps and regions that are either unfinished or incorrectly assembled, stemming from the utilization of BAC clones from different individuals. Consequently, approximately 200 megabase pairs (Mbp) are unavailable for DNA sequence-based analyses, and 230 Mbp are inaccurately represented in the GRCh38 genome. The T2T Consortium, through the use of the uniformly homozygous CHM13hTERT cell line, successfully overcame these limitations and released the initial draft of the T2T-CHM13 reference assembly in 2019, with the most recent version T2T-CHM13v2.0 released in 2022. By employing a combination of various sequencing techniques, the T2T Consortium achieved the sequencing of the 8% of the human genome that was absent in the GRCh38 assembly. This breakthrough led to the identification of 1956 novel predicted genes, with 99 of them predicted to be protein-coding [1]. The discovery of the novel MUC3B gene using the T2T-CHM13 assembly and its subsequent experimental validation [3] represents an initial demonstration of the breadth and utility of the T2T-CHM13 assembly.

The T2T-CHM13 assembly presents novel opportunities in human disease research. The T2T Consortium demonstrated that analysis of challenging, clinically relevant genes could be enhanced by a factor of 12 This was primarily achieved through the refinement of the sequences of 70 protein-coding genes that were previously plagued by falsely collapsed or duplicated regions. The reduction of false-positive variant calls was most pronounced in analyses conducted with Illumina short-read sequencing data [4]. This suggests that a multitude of putative disease-causing candidate genes and genetic variants may have been identified under erroneous assumptions. The impact of this on the discovery of disease-relevant genes from transcriptome data remains to be explored.

The T2T-CHM13 assembly encompasses an additional 9.9 Mbp of rDNA sequence, enabling the creation of an atlas of ribosomal RNA (rRNA) variation in both health and disease, with a particular focus on their involvement in cancer [5]. Ribosome biogenesis is implicated in numerous human conditions [6] including neurodegenerative disorders like Alzheimer’s [7] and Parkinson’s disease [8]. To assess the potential of the T2T-CHM13 assembly in the discovery of candidate genes for human neurological disorders, multiple RNA sequencing (RNA-seq) datasets were remapped to both the T2T-CHM13 and the GRCh38 genome assemblies in a comparative pipeline. This led to the identification of 336 novel candidate genes associated with eight distinct neurological pathologies.

## Results

The NCBI sequence read archive was keyword searched for RNA-seq datasets pertaining to human neurological conditions. This yielded the selection of 26 eligible studies, of which 17 datasets resulted in the discovery of differentially expressed genes (DEGs). These genes were not annotated in the GRCh38.p14 genome assembly but were exclusively identified in the T2T-CHM13v2.0 assembly, referred to as novel genes in the following text. Table 1 presents a summary of the datasets that produced novel candidate genes for human neurological pathologies, along with their respective abbreviations (comprehensive dataset information can be found in Additional file 1). No novel genes were detected in datasets from Parkinson’s disease, alcoholism, and mental illness studies.

**Table 1:** RNA-seq datasets from studies on human neurological disorder, which yielded novel candidate genes with the T2T-CHM13 assembly

Disorder	Experimental design	Abbreviation	N (ctrl vs case)	Publication
Alzheimer’s disease	Alzheimer’s patient’s induced neurons	AD/n	8;10	[9]
Amyotrophic lateral sclerosis	Single-cell SOD1 E100G ALS iPSC-derived motor neurons	ALS/sc	192;192	[10]
Amyotrophic lateral sclerosis	Patient iPSC-derived motor neurons harboring SOD1 mutations	ALS/mn	3;2	[11]
Amyotrophic lateral sclerosis	CRISPR/Cas9-mediated targeted gene correction in patient iPSCs	ALS/crispr	2;2	[12]
Autism spectrum disorder	NSCs derived from patient fibroblasts	ASD/nsc	5;8	[13]
Autism spectrum disorder	Postmortem brains	ASD/pb	2;2	[14]
Epilepsy	Dentate granule cells from hippocampi from patients with mesial temporal lobe epilepsy	E/dc	14;8	[15]
Epilepsy	Patient primary skin fibroblasts	E/pf	5;6	[16]
Epilepsy	Patient-derived neuronal cells	E/nc	3;3	[17]
Epilepsy	Patient-derived neuronal astrocytes	E/na	3;3	[17]
Epilepsy	Patient-derived neuronal oligodendroglial progenitor cells	E/nopc	3;3	[17]
Glioblastoma	Endothelial cells isolated from post-mortem and resection surgery from human cortex	Gliob/ec	7;5	[18]
Glioma	Patient-derived glioma	Glio	6;16	[19]
m.3242 A > G mtDNA mutation	Pateient fibroblasts	mtDNA/m.3243	6;6	[20]
Multiple sclerosis	Patient CD4 + T cells	MS/CD4	5;5	[21]
Multiple sclerosis	Patient CD19 + B cells	MS/CD19	6;6	[21]
Multiple sclerosis	Patient white matter lesions	MS/bl	25;72	[22]

**Fig. 1:**
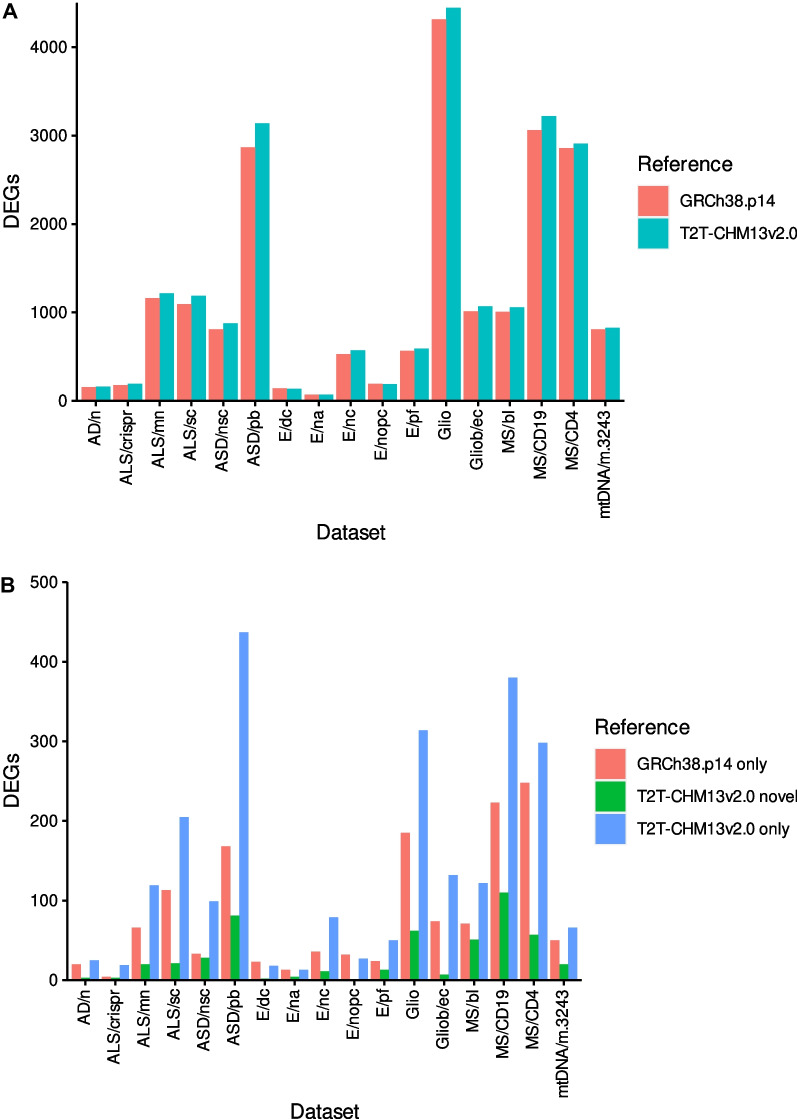
Differential expression analysis results of RNA-seq datasets from studies on human neurological disorders. **A** Numbers of differentially expressed genes (DEGs) discovered with the GRCh38.p14 and the T2T-CHM13v2.0 genome assemblies. **B** Numbers of DEGs, which were discovered only with the GRCh38.p14 or the T2T-CHM13v2.0 genome assembly as well as numbers of novel DEGs, which were not annotated in GRCh38.p14

The number of significant DEGs (abs. Log_2_ FC > 1, *p*-adj. < 0.01) varied between 72 and 4315 when utilizing the GRCh38.p14 assembly and between 72 and 4444 with the T2T-CHM13v2.0 assembly (Fig. 1A). In all but three studies, the number of discovered DEGs was higher with the T2T-CHM13v2.0 assembly. On average, 8.4% of the genes discovered with the GRCh38.p14 assembly were not detected using the T2T-CHM13v2.0 assembly, whilst conversely, this percentage increased to 12.1% undetected genes. Among all studies, a total of 1279 different DEGs were exclusively identified using the GRCh38.p14 assembly (Additional file 2). These include genes that are challenging to assemble, i.e. ten micro RNAs (miRNAs), 13 HLA genes, 29 zinc-finger proteins, 30 long noncoding RNAs (lincRNAs), and 259 genes of uncertain function. However, it should be noted that a considerable number of these genes were close to the significance thresholds. In particular, among all studies 605 of the DEGs discovered only with the GRCh38.p14 assembly exhibited an abs. Log_2_ FC > 0.9 and *p*-adj < 0.015 when mapped to the T2T-CHM13v2.0 assembly. This suggests that 778 DEGs identified with the GRCh38.p14 assembly were potential false positives, averaging 3.6% across all studies. As no universally accepted consensus regarding significance thresholds in RNA-seq studies exists, a volcano plot depicting potential false-positive DEGs is provided in Additional file 3 to illustrate the statistical distribution. The underlying cause of potential false-positive DEG detection was further investigated using the three DEGs with the lowest Log_2_ FC among the T2T-CHM13v2.0 mappings (*POTEI*, *FAM227B*, and *LOC105375228*).

The *POTEI* gene (position in T2T-CHM13v2.0: Chr2:130.893.363–130.943.575) was significantly differentially expressed (DE) in the dataset MS/CD19 (Log_2_ FC = 1.266 in GRCh38.p14, Log_2_ FC = 0.0376 in T2T-CHM13v2.0). Remarkably, 73% of the reads mapped to the gene in GRCh38.p14 failed to map to the same gene using the T2T-CHM13v2.0 assembly. Instead, these reads were mapped to the genes *ACTB* (position in T2T-CHM13v2.0: Chr7: 5.644.671–5.648.124) and the *POTEE* gene (position in T2T-CHM13v2.0: Chr2: 131.643.505–131.702.729). Both *POTEE* and *POTEI* belong to the ankyrin domain family, a group of genes known for containing repetitive regions. *FAM227B* was identified in the E/pf dataset (Log_2_ FC = 1.675 in GRCh38.p14, Log_2_ FC = −0.007 in T2T-CHM13v2.0), and *LOC105375228* was detected in the ALS/sc dataset (Log_2_ FC = −1.470 in GRCh38.p14, Log_2_ FC = −0.072 in T2T-CHM13v2.0). The number of reads mapped to the genes were similar for both genes in both alignments, with discrepancies of 0.17% and 1% between mapped reads. Differences in the intron/exon structures of the genes between the two genome assemblies accounted for the differences in the DE results. Overviews of mapped reads against these three regions to both respective genome assemblies can be found in Additional file 4.

**Fig. 2:**
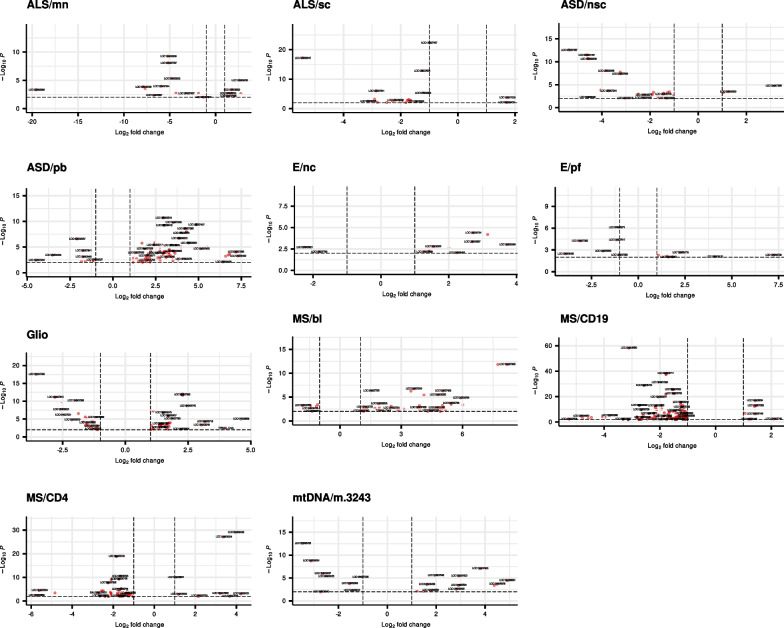
Volcano plots of novel differentially expressed genes (DEGs) from datasets yielding more than ten novel DEGs

**Fig. 3:**
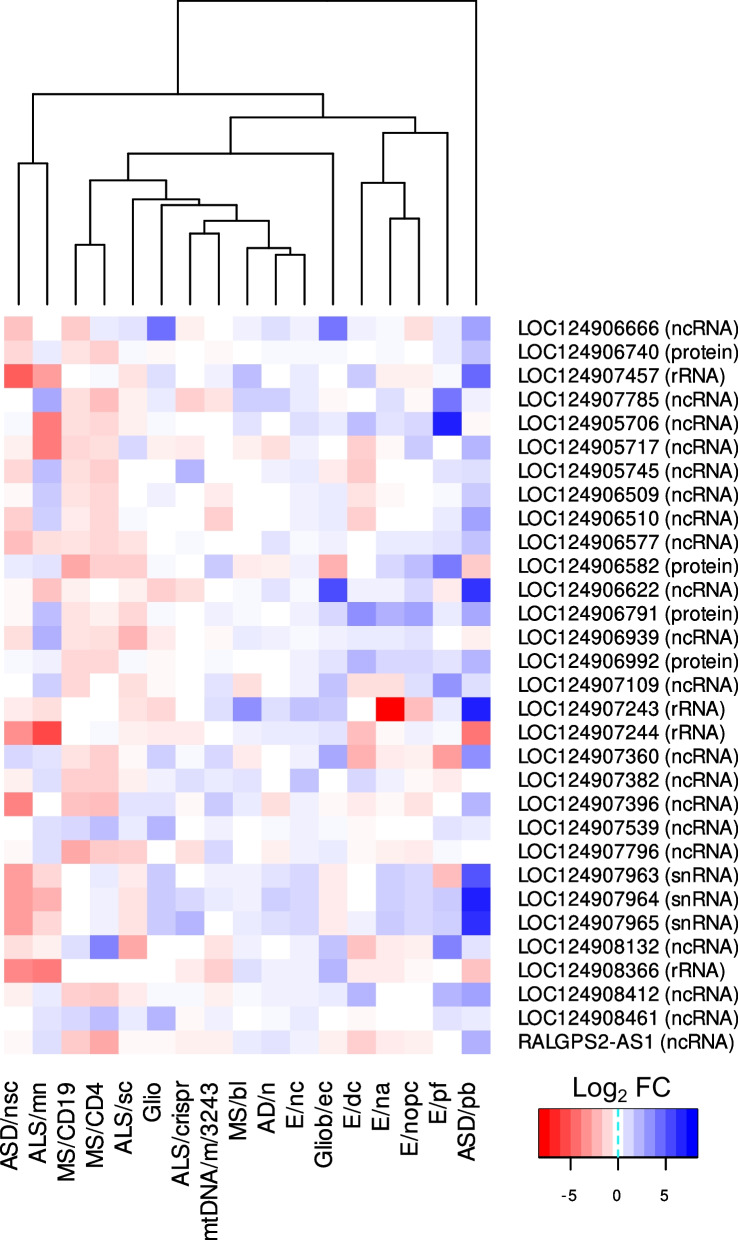
Heatmap of novel genes which were differentially expressed in at least three datasets. Log_2_ fold changes of cases vs. controls are displayed

The amount of novel genes that were DE in the investigated conditions ranged from 1 to 110, constituting an average of 2.3% of all DEGs (Fig. 1B, Additional file 1). The DE results for all novel genes significantly associated with the conditions under investigation are summarised in Additional file 5, while Additional file 6 provides an overview of all significant DEGs identified using the T2T-CHM13v2.0 assembly, including those exclusively detected with the GRCh38.p14 assembly. Concordant and discordant DEGs between the reference assemblies are presented in the form of volcano plots in Additional file 7. Figure 2 illustrates volcano plots for datasets yielding more than ten novel potential pathology related genes. To identify novel genes that might play a role in multiple conditions, a heatmap of genes that where DE in at least three of the datasets under investigation was generated (Fig. 3). This analysis led to the discovery of 31 genes in total, comprising 20 ncRNAs, four proteins, four rRNAs, and three snRNAs (Additional file 8).

**Fig. 4:**
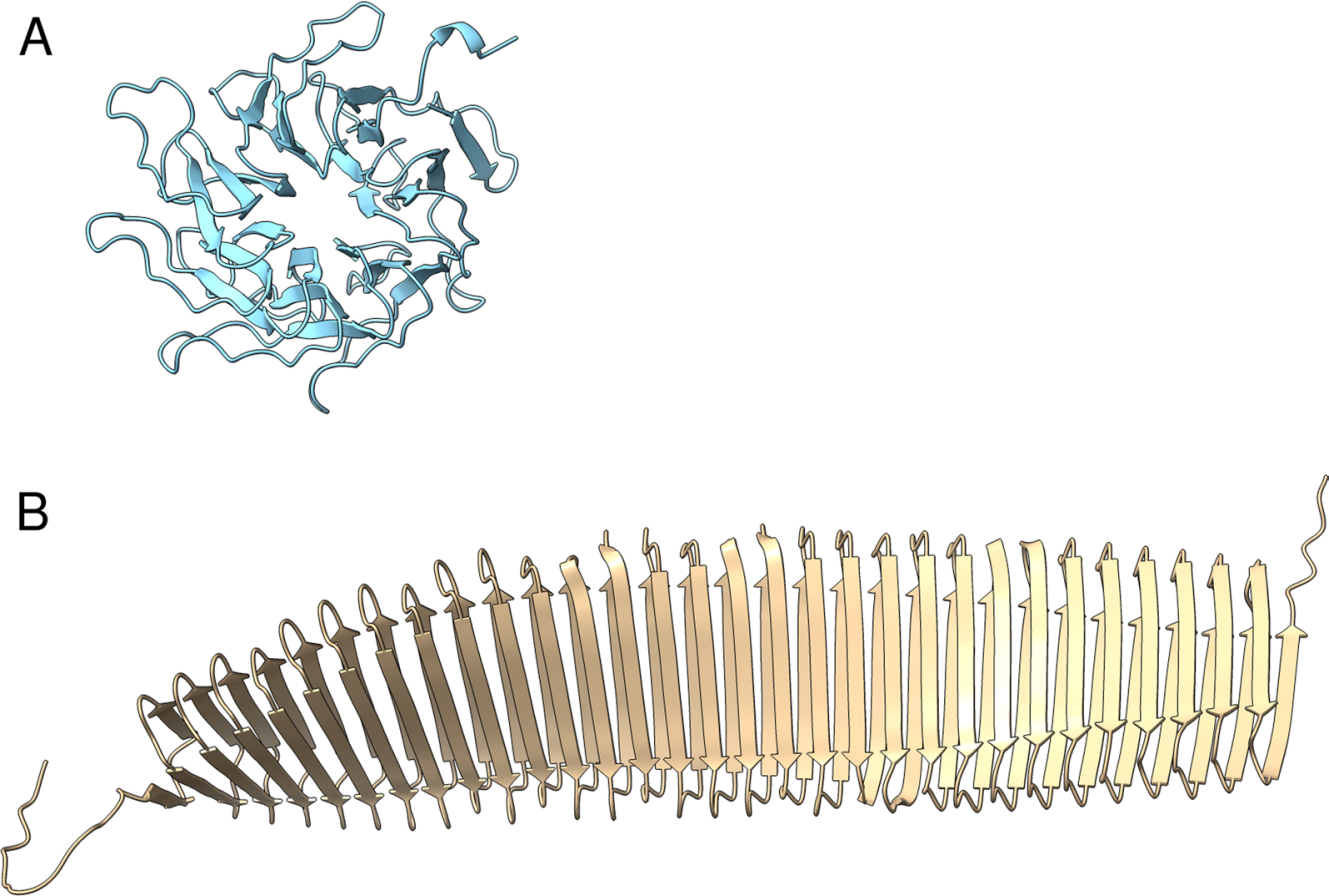
Protein structure prediction results with AlphaFold for the novel proteins **A** LOC124906791 and **B** LOC124906582

Protein secondary structure prediction using Alphafold was carried out for the sequences of the novel proteins LOC124906582, LOC124906740, LOC124906992, and LOC124906791 (Fig. 4). The predicted scores from the local distance difference test (pLDDT) for LOC124906740 and LOC124906992 were below 50, indicating a low level of confidence. Consequently, these protein structures were not further considered.

The transcript of the pore-like structured protein LOC124906791 (Fig. 4A) was significantly upregulated (*p*-adj. < 0.01), approximately 2.7-fold, in the E/na, E/nc, and, E/nopc datasets. Additionally, it showed upregulation (*p* < 0.05) ranging from 2.1 to 3.6-fold in the ASD/pb, E/dc, and ALS/mn datasets. In contrast, LOC124906582 was significantly downregulated, approximately twofold in the ASD/pb, Gliob/ec, and MS/CD19 datasets. It also showed a 1.5-fold downregulation in the MS/CD4 dataset, but an upregulation ranging from 1 to 4-fold in the E/na, E/nc, E/nopc, and E/pf datasets.

The gene product was predicted to be a highly ordered, cross-beta strand-rich tubular protein (Fig. 4B), resembling the structural features seen in the cryo-electron microscopy images of fibrillary amyloid-β(1–42) [23]. Between its N-terminal amino acid sequence ‘MTKIRRSSNNNSYMSS’ and its C-terminal sequence ‘LQQLWQEE’ the 801 aa protein consists of the highly repetitive motive AASAVAVAVAGGV, with slight sequence variation, consisting of the amino acids A (382; 49%), V (246; 32%), G (108; 14%), S (36; 5%), I (2; 0%), T (2; 0%), and L (1; 0%). To predict its propensity to aggregate AlphaFold was used. The predictions of dimer, trimer, and tetramer structures of LOC124906582 can be found in Additional file 9. An amyloid typical cross β-sheet structure [24] was predicted in all three types of multimers. Since these simulations are computationally highly demanding, no higher-order multimers could be predicted.

## Discussion

The release of the first complete sequence of a human genome by the T2T Consortium unlocked previously hidden genomic regions for genetic analyses and exposed previously misassembled sequences. To assess its potential in discovering novel genes associated with human neurological disorders, multiple publicly available RNA-seq datasets were analysed using the T2T-CHM13 assembly, comparing it to the GRCh38 assembly. Through this approach, 336 candidate genes for eight neurological disorders that were not annotated in the GRCh38 assembly were discovered. The subsequent sections discuss the results for each of the disorders, with a particular focus on the implications of this study for future research. Due to the extensive number of novel genes discovered in this study, the discussion will primarily concentrate on the most significant findings or functionally related gene groups. It is important to note that this comparative study relies solely on transcriptome data. Therefore, no final conclusions about the functions of these novel genes can be drawn. Consequently, the following paragraph should be viewed as potential directions for future experimental research aimed at characterising these findings functionally.

### Alzheimer’s disease

Among the three novel genes identified in induced neurons derived from Alzheimer’s disease patients was the gene *LOC124906857*, which encodes phosphoglucomutase-like protein 5. Phosphoglucomutase 5 is predominantly found in adherens junctions, which are believed to be involved in blood–brain barrier (BBB) permeability regulation in Alzheimer’s disease [25]. Hence, the upregulation of *LOC124906857* may lead to higher BBB permeability and an influx of neurotoxic plasma-derived components, cells, and pathogens [26]. This suggests a potential modulating role for *LOC124906857* in the disease, which should be validated in a suitable experimental model system.

### Amyotrophic lateral sclerosis

Novel candidate genes for amyotrophic lateral sclerosis (ALS) were revealed, which included eight rRNAs and two spliceosomal RNAs, all of which were downregulated. Protein synthesis [27] and spliceosomal deficiencies [28] have been linked to ALS pathology. The interruption of ribosomal translation results in the binding of the SURF complex to the exon junction, triggering mRNA decay [29]. SMG1 is one of the four SURF components, and the *LOC124907829* gene, encoding the novel protein *serine/threonine-protein kinase SMG1-like*, is upregulated in motor neurons derived from ALS patients. This may lead to mRNA decay in ALS patient-derived motor neurons and presents a link to the nonsense-mediated mRNA decay reported by Xu et al. [30] in animal and cellular models of ALS. The differential expression of *LOC124907841*, which encodes bolA-like protein 2, points towards oxidative stress [31], a potential therapeutic target in ALS therapy [32]. To validate its relevance to ALS pathology, further investigation into the involvement of *LOC124907841* in the clearance of reactive oxygen species is warranted.

### Autism spectrum disorders

Of the two datasets originating from autism spectrum disorder (ASD) studies, the ASD/pb dataset had a sample size of only two individuals per group. Therefore, one must assume low statistical power for the ASD/pb dataset, and consequently, I will only discuss overlapping genes with the ASD/nsc dataset and highly significant DEGs. Among the common genes uncovered are three U2 spliceosomal RNA encoding genes. The study from which the ASD/pb dataset was derived describes dysregulation of RNA editing in the brains of autistic individuals [14] and it is reasonable to assume, that these novel spliceosomal RNAs are involved. However, it is worth noting that the transcripts encoding those genes were upregulated in post-mortem brains but downregulated in neuronal stem cells (NSCs). This suggests dysregulation in their expression during development and adulthood. Analysis mistakes regarding the two datasets were excluded, so the observed opposite direction of differential expression of novel genes between the two datasets is most likely an effect of the developmental stage of the sequenced tissue/cells. However, a sample mix-up in one of the studies cannot be excluded, so these results should be interpreted with caution.

Among the ten most significant novel genes observed in the ASD/pb dataset is *LOC124905679*, which was predicted to encode hornerin, a gene previously linked to ASD on the genomic level [33]. Additionally, the tektin-4-like gene *LOC124907385* was upregulated in the ASD/nsc dataset. A missense variant in *TEKT4* was discovered in a family with PSMD12 haploinsufficiency, a neurodevelopmental disorder with autistic features.

Analysis of both ASD studies also identified two ribosomal RNAs, *LOC124907244* and *LOC124907457*. Ten rRNAs were DE in both studies. Recently, the ribosomes have gained central importance in understanding the development of ASD [34]. Mutations in genes relevant for translation control, such as *FMR1*, *TSC2*, and *PTEN*, have shown high penetrance in ASD development [35]. Therefore, the ten novel rRNA encoding genes discovered here are strong candidates for being downstream effectors of these translation control genes.

### Epilepsy

Regarding the novel candidate genes uncovered in the study presented here, epilepsy stands out as the most intriguing neurological disorder. The transcript of *LOC124906791*, a protein with pore-like structured and rich in beta strands (Fig. 4A), was significantly upregulated in four epilepsy datasets (E/dc, E/na, E/nc, and E/nopc). Given that epilepsy primarily involves disruptions in ion exchange homeostasis, this putative membrane pore-forming protein becomes of paramount interest for future epilepsy research. Its upregulation in all four studies suggests that increased expression of the protein may lead to ion exchange with the extracellular space, a major trigger for epileptic seizures [36]. The role of pore-forming proteins in epilepsy has only recently come under discussion [37] and the evidence provided here, positions LOC124906791 as a promising candidate for the development of inhibitory drugs.

Another novel protein identified, LOC124906582, predicted to have a highly ordered structure rich in cross beta strands, with a tubular shape resembling the structure of fibrillary amyloid-β(1–42) [23], was also significantly upregulated in four epilepsy datasets (E/na, E/nc, E/nopc, and E/pf). AlphaFold simulations demonstrated the ability of LOC124906582 to form dimeric, trimeric, and tetrameric multimers, suggesting its capacity to create aggregates. Compelling evidence for the involvement of amyloidogenic proteins like amyloid-β, α-Synuclein, and Tau in the development of late-onset epilepsy is accumulating [38, 39], underscoring the significance of the newly discovered protein LOC124906582 as a hitherto unknown key player in epilepsy development. Therefore, conducting in vitro co-aggregation studies with the aforementioned amyloidogenic proteins would offer valuable evidence for a more comprehensive experimental validation of this novel protein.

### Glioma

The term glioma encompasses a group of cancer types that affect glial cells in the brain. Glioblastoma is a grade 4 type and the most aggressive form of glioma. This study unveiled 62 novel candidate genes for glioma and 7 novel candidate genes for glioblastoma. Among the top 20 novel genes associated with glioma, based on the lowest *p*-values, 17 are ncRNAs. Numerous studies have linked ncRNAs to glioma pathology, and they were connected to poor patient survival rates. Hence, they serve as predictive markers of disease progression [40]. The newly discovered ncRNAs should be correlated with disease phenotypes to enhance predictions of patient outcomes.

One of the top 20 DEGs is *LOC124907389*, which encodes the leucine-rich repeat transmembrane protein FLRT2. In breast cancer, *FLRT2* has been identified as a tumor suppressor gene [41], and interestingly, *LOC124907389* exhibited a twofold downregulation in patient-derived glioma tissue. This suggests that this novel *FLRT2*-like gene may also exert tumor suppressive functions in relation to glioma.

### Multiple sclerosis

The analysis of three distinct multiple sclerosis (MS) datasets—post-mortem white matter lesions, CD4^+^ T cells, and CD19^+^ T cells – has unveiled 146 novel potential disease-associated candidate genes. It is noteworthy that the MS/bl dataset included an exceptionally high number of cases (*N* = 72). The DE ncRNAs *LOC124907785* and *LOC124907382* were common across all datasets. These ncRNAs were upregulated in brain lesions but downregulated in immune cells, suggesting a significant role in MS pathology. A blast search revealed that all four annotated *LOC124907785* transcripts exhibited a sequence identity ranging from 78 to 86% with multiple transcripts of *LOC124906734*, encoding the protein translation initiation factor IF-2-like. This gene was notably downregulated in CD4^+^ cells and slightly downregulated in CD19^+^ cells. A homozygous missense variant in the *EIF2B2* (eukaryotic Translation Initiation Factor 2B Subunit Beta) gene has been identified as causative for early-onset vanishing white matter disease [42]. This finding strengthens the case for LOC124907785, a putative inhibitory ncRNA differentially expressed in all three MS datasets, as a strong candidate for a regulatory RNA with disease-modifying properties.

Additionally, four other ncRNAs were DE in both brain lesions and CD19^+^ T cells. Three of these ncRNAs (LOC124905722, LOC124906211, and LOC124907546) were upregulated in brain lesions but downregulated in immune cells, while one (LOC124907109) was downregulated in both datasets. ncRNAs have been a subject of intense research in recent MS studies [43–46]. The observed pattern of upregulation in brain lesions and downregulation in immune cells suggests a disturbed regulation of ncRNA expression in MS.

A total number of 51 novel DEGs were discovered in MS patient brain lesions, with more than 50% highly upregulated rRNA-encoding genes (rDNA, avg. Log_2_ FC = 4.6). Since this data was produced with an rRNA removal step [22], and elevated rDNA levels were only detected in the 72 cases, contamination with rRNA can be excluded. Spurlock et al. reported elevated levels of misprocessed rRNA in mononuclear cells from individuals with relapsing remitting multiple sclerosis, attributing this to environmental factors rather than genetics [47]. Both findings therefore strongly suggest a significant role for ribosomes in MS pathology.

### Mitochondrial DNA mutation m.3243 A > G

One of the most prevalent mitochondrial DNA (mtDNA) mutations is m.3243 A > G [48], which manifests with a broad spectrum of clinical features, including seizures, stroke-like episodes, hearing impairment, gastrointestinal disturbance, psychiatric involvement, ataxia [49], and neurodegeneration [50]. Among the newly discovered genes which were DE in patient-derived cell lines, 13 were ncRNAs and six were proteins. The novel *LOC124907531* gene, encoding amyloid-beta A4 precursor protein-binding family A member 2-like, demonstrated a threefold upregulation. The protein product of the *APBA2* gene interacts with the amyloid precursor protein (APP) and influences the proteolytic production of amyloid-β [51]. The neurodegenerative features of m.3243 A > G cases have been attributed to defects in nitric oxide metabolism and mtDNA-related mitochondrial respiration [50]. These are also features of Alzheimer’s disease and other neurodegenerative disorders [52]. Although the m.3243 A > G mutation could not be linked to cases of early-onset Alzheimer’s disease [53], an involvement of amyloid-β in m.3243 A > G linked neurodegeneration has not been ruled out yet and should be investigated in connection to amyloid-β pathology.

### Implications of the study

The data presented here indicate that about half of the 26 selected studies on neurological disorders have yielded novel candidate genes with differential expression for further study. Furthermore, on average 3.6% of the DEGs discovered with the GRCh38 genome assembly did not exhibit differential expression when reads were aligned to the T2T-CHM13 assembly. This suggests that prior analyses were hindered by the identification of putative false-positive DEGs caused by inaccuracies in the GRCh38 assembly. In light of these findings, it is highly recommended to re-map RNA-seq data from older studies to validate the integrity of the published data. Additionally, this approach can help identify if any phenotype-associated genes are among the 1956 novel genes discovered by the T2T Consortium. The discovery of many DEGs presented here was only possible by unlocking previously inaccessible regions of the human genome. Notably, the highly repetitive epilepsy-associated protein-coding gene *LOC124906791* was previously obscured by technical limitations, and the revelation of numerous rRNA encoding transcripts upregulated in white matter lesions of MS patients was only made possible through the assembly of an additional 9.9 Mbp of rDNA regions in the T2T-CHM13 assembly. While all the datasets examined here were based on Illumina short reads, the use of long read RNA sequencing techniques [54] in conjunction with the T2T-CHM13 assembly or even a human pangenome [55] promises significant improvements in the quality and depth of future human transcriptome analyses.

## Conclusions

The T2T-CHM13 assembly has the potential to unravel the full potential of RNA-seq studies focused on the discovery of candidate genes for human disorders. A total number of 336 novel, previously inaccessible genes, were linked to eight different neurological conditions, which provides substantial rationale for their validation in suitable experimental model systems. Past results generated with the GRCh38 assembly should be interpreted with caution and validated with the T2T-CHM13 assembly. A discordance of 3.6% in significantly associated DEGs between the two reference assemblies, raises the question as to the extent false-positive gene-disease associations have been a source of bias in past transcriptome studies based on the GRCh38 assembly. The research strategy outlined here should be adapted to different fields of human research, to expand the repertoire of disease-associated genes for a better understanding and ultimately treatment of medical conditions.

## Methods

### Data acquisition

The NCBI sequence read archive (SRA) was keyword searched for RNA-seq data on human neurological conditions. Datasets were bulk downloaded with JDownloader2 and transferred to the GWDG (Gesellschaft für wissenschaftliche Datenverarbeitung mbH Göttingen) high performance computing (HPC) cluster.

### Differential gene expression analysis

Quality control and trimming of raw sequencing reads were performed with Trimmomatic version 0.36 (settings: PE -phred33 LEADING:3 TRAILING:3 SLIDINGWINDOW:4:15 MINLEN:36) [56]. The reference assemblies used for RNA-seq read alignment were GRCh38.p14, NCBI assembly GCF_000001405.40, annotation release 110 and T2T-CHM13v2.0, NCBI assembly GCF_009914755.1, annotation release 110. Splice sites were derived from the General Transfer Format (GTF) files and reads were aligned with HISAT2 (Version 2.1.0) [57] using default settings. FeatureCounts from the Subread package (Version 2.0.0) was used to count exon spanning reads [58]. DE analyses were conducted with DESeq2 (Version 1.40.1) [59]. DEGs with a *p*-adj. < 0.01 were considered significant and DEGs with a *p*-value < 0.05 were considered possibly biological relevant. Adjusted p-values were pre-corrected for multiple testing based on the false discovery rated according to Benjamini and Hochberg, which is implemented in DESeq2 (see DESeq2 documentation for further information). Data plots were produced with RStudio (Version 2023.03.0 Build 386) and ggplot2 (Version 3.4.2). Volcano plots were created with the R package EnhancedVolcano (Version 1.18.0). Concordance and discordance between datasets was computed with the R package VennDiagram (Version 1.7.3).

### Protein secondary structure prediction

Monomeric protein structures were predicted with AlphaFold 2.3.2 [60] with default settings. Multimeric proteins were predicted with Alphafold using the *–model_preset* = *multimer* flag. The structure predictions with the highest model confidence (ranked_0) were used for further analysis. AlphaFold was run on a HPC cluster node with the following specifications: 2 × Cascade Lake Intel Gold 6252 CPU (24 threads at 2.1 GHz), 2 × NVidia Tesla V100 (32 GB memory), 384 GB RAM. Protein structures were visualised with ChimeraX (Version 1.6.1) [61]. Quality metrics of protein structure predictions (predicted local distance difference test (pLDDT), per-structure quality estimation scores (pTM), interface pTM score (ipTM), and predicted aligned error (PAE)) are summarised in Additional file 10.

### Supplementary Information

**Additional file 1**. Complete information on the RNAseq studies used for analyses.

**Additional file 2**. Genes which were differentially expressed when mapped to GRCh38.p14 assembly but not when mapped to the T2T-CHM13v2.0 assembly.

**Additional file 3**. Differentially expressed genes, which were identified with the GRCh38.p14 genome assembly but not with the T2T-CHM13v2.0 assembly. Log2 fold changes and adjusted *p* values of differential expression analysis with the T2T-CHM13v2.0 assembly are shown.

**Additional file 4**. Genomic regions of the putative false positive DEGs *POTEI*, *FAM227B*, and *LOC105375228* viewed with the Integrative Genomics Viewer.

**Additional file 5**. Differential expression results for novel genes discovered in the analyses of neurological disorders.

**Additional file 6**. Complete results of differential expression analysis with the T2T-CHM13v2.0 assembly including genes, which were only discovered using the GRCh38.p14 assembly.

**Additional file 7**. Volcano plots of differential expression analysis with the T2T-CHM13v2.0 assembly, including genes, which were only discovered using the GRCh13.p14 assembly.

**Additional file 8**. Batch entrez results for novel genes, which were differentially expressed in at least three studies.

**Additional file 9**. Dimer (**A**), trimer (**B**), and tetramer (**C**) structure prediction of the LOC124906582 protein with Alphafold.

**Additional file 10**. Alphafold quality metrics of predicted protein structures: predicted local distance difference test (pLDDT), per-structure quality estimation scores (pTM), interface pTM score (ipTM), and predicted aligned error (PAE).

## Data Availability

Raw sequencing data was acquired from the European Nucleotide Archive. The BioProject IDs of the studies are PRJEB19652, PRJEB23143, PRJEB30906, PRJEB44542, PRJEB89471, PRJNA236453, PRJNA290212, PRJNA376020, PRJNA421728, PRJNA483174, PRJNA563467, PRJNA576512, PRJNA589589, PRJNA589589, PRJNA589589, PRJNA604108, PRJNA670696, and PRJNA732455.

## Abbreviations

ALS: Amyotrophic lateral sclerosis
ASD: Autism spectrum disorder
BBB: Blood–brain barrier
DE: Differentially expressed
DEGs: Differentially expressed genes
Mbp: Mega base pairs
MS: Multiple sclerosis
mtDNA: Mitochondrial DNA
NSCs: Neuronal stem cells
RNA-seq: RNA sequencing
rDNA: rRNA encoding DNA
rRNA: Ribosomal RNA
T2T: Telomere-to-Telomere
