# Supplementary material for: Transcriptome driven discovery of novel candidate genes for human neurological disorders in the telomer-to-telomer genome assembly era
Source: Hum Genomics. 2023 Oct 23;17:94. doi: 10.1186/s40246-023-00543-y (PMC10594789; doi:10.1186/s40246-023-00543-y)

Additional File S3: Differentially expressed genes, which were identified with the GRCh38.p14 genome assembly but not with the T2T-CHM13v2.0 assembly. Log_2 fold changes and adjusted p values of differential expression analysis with the T2T-CHM13v2.0 assembly are shown.

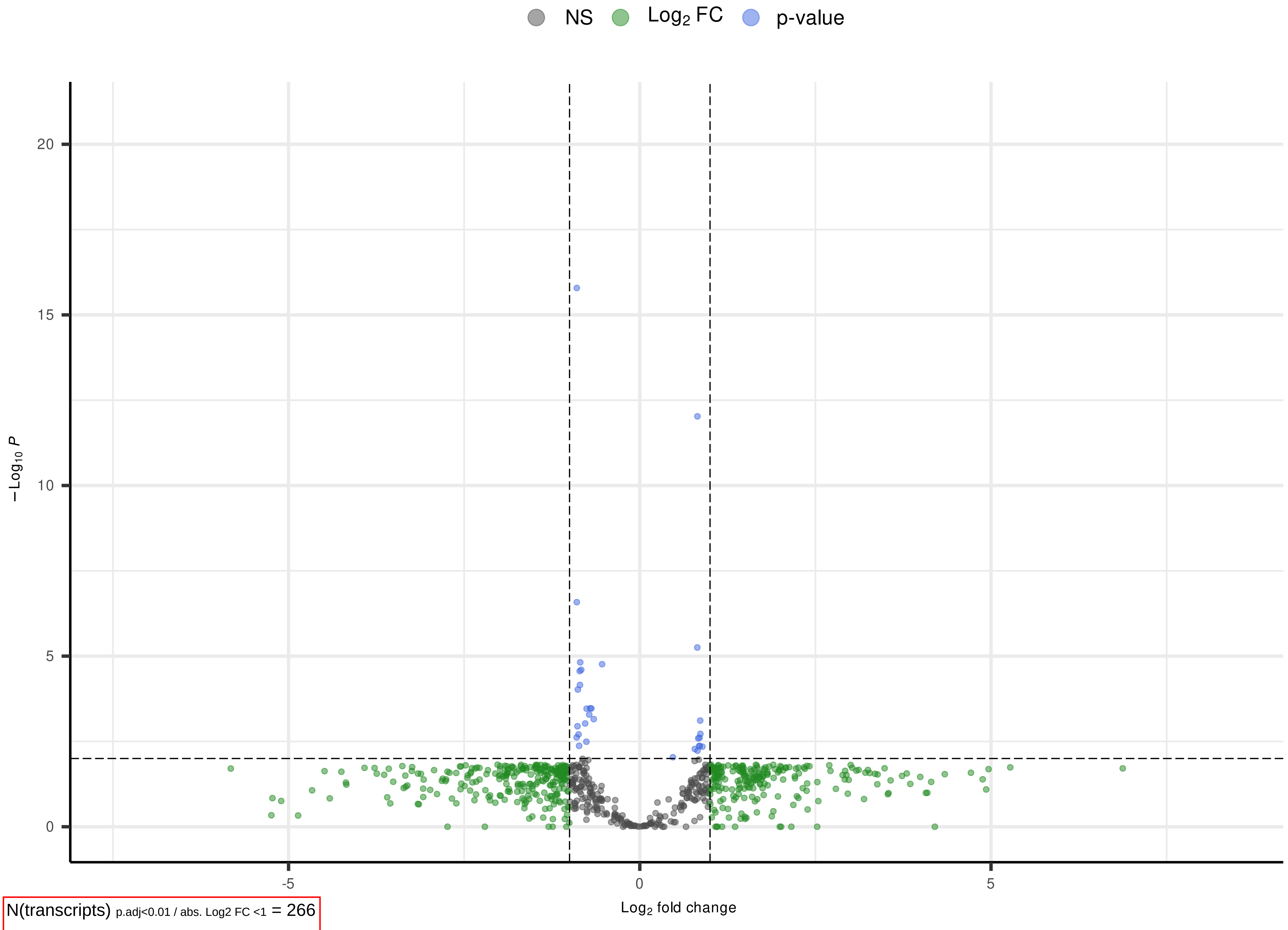

Supplement: Supplementary file 3 — Additional file 3. Differentially expressed genes, which were identified with the GRCh38.p14 genome assembly but not with the T2T-CHM13v2.0 assembly. Log2 fold changes and adjusted p values of differential expression analysis with the T2T-CHM13v2.0 assembly are shown. [file 40246_2023_543_MOESM3_ESM.pdf]
