# Supplementary material for: Transcriptome driven discovery of novel candidate genes for human neurological disorders in the telomer-to-telomer genome assembly era
Source: Hum Genomics. 2023 Oct 23;17:94. doi: 10.1186/s40246-023-00543-y (PMC10594789; doi:10.1186/s40246-023-00543-y)
Supplement: Supplementary file 4 — Additional file 4. Genomic regions of the putative false positive DEGs POTEI, FAM227B, and LOC105375228 viewed with the Integrative Genomics Viewer. [file 40246_2023_543_MOESM4_ESM.pdf]

Additional File S4: Genomic regions of the putative false positive DEGs *POTEI*, *FAM227B*, and *LOC105375228* viewed with the Integrative Genomics Viewer.

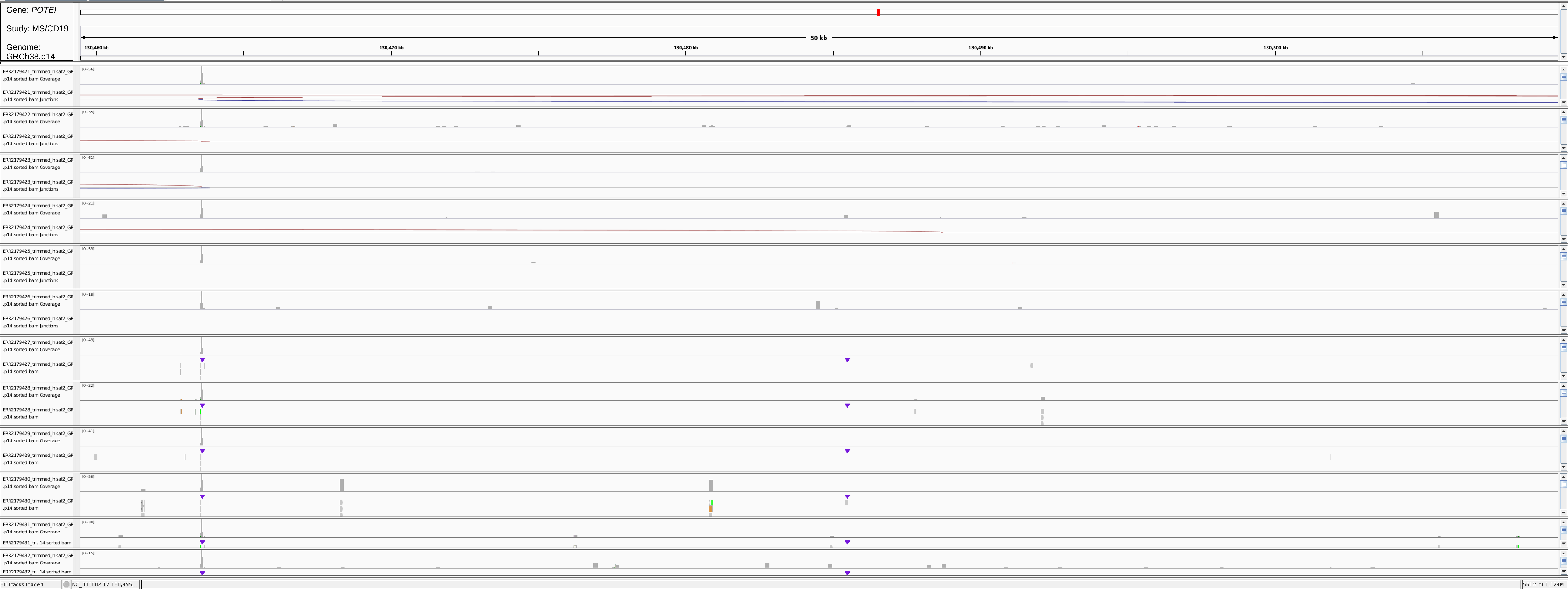

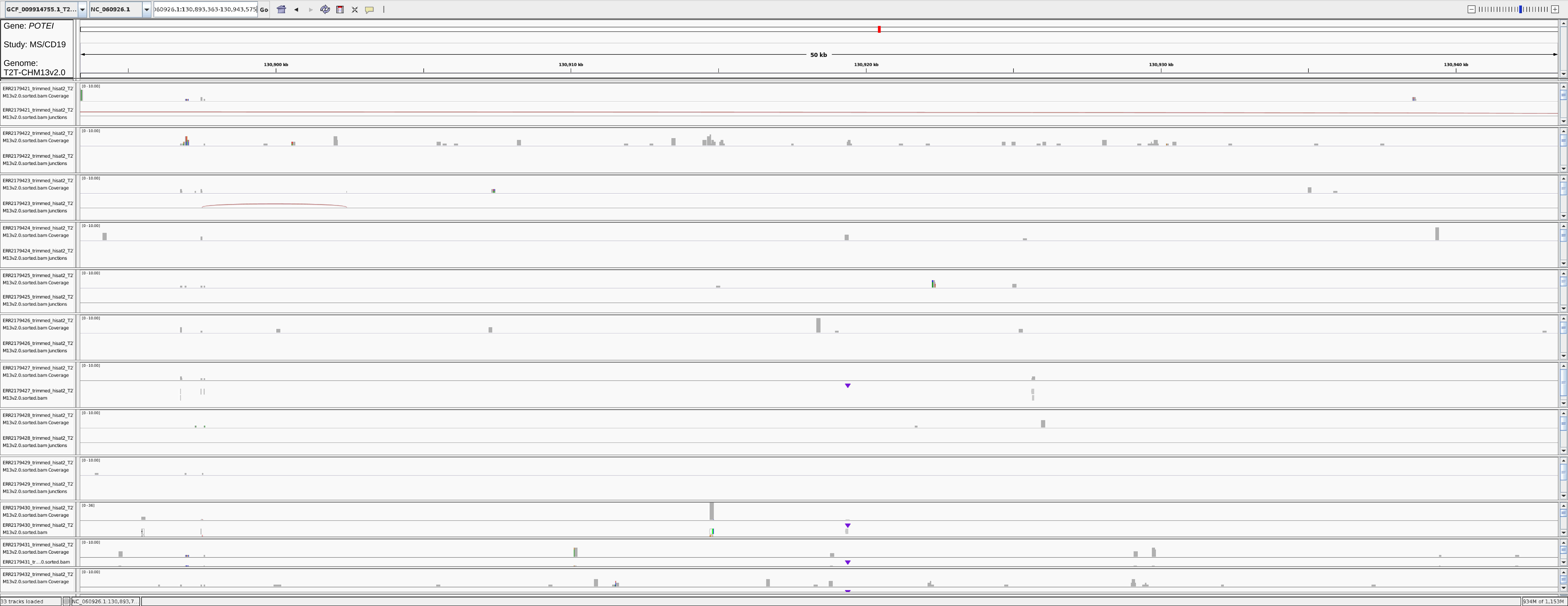

GCF_009914755.1_T2...
NC_060926.1
060926.1:130,893,363-130,943,575
Go
Gene: POTEI
Study: MS/CD19
Genome: T2T-CHM13v2.0
50 kb
130,900 kb
130,910 kb
130,920 kb
130,930 kb
130,940 kb
ERR2179421_trimmed_hisat2_T2 M13v2.0.sorted.bam Coverage
ERR2179421_trimmed_hisat2_T2 M13v2.0.sorted.bam Junctions
ERR2179422_trimmed_hisat2_T2 M13v2.0.sorted.bam Coverage
ERR2179422_trimmed_hisat2_T2 M13v2.0.sorted.bam Junctions
ERR2179423_trimmed_hisat2_T2 M13v2.0.sorted.bam Coverage
ERR2179423_trimmed_hisat2_T2 M13v2.0.sorted.bam Junctions
ERR2179424_trimmed_hisat2_T2 M13v2.0.sorted.bam Coverage
ERR2179424_trimmed_hisat2_T2 M13v2.0.sorted.bam Junctions
ERR2179425_trimmed_hisat2_T2 M13v2.0.sorted.bam Coverage
ERR2179425_trimmed_hisat2_T2 M13v2.0.sorted.bam Junctions
ERR2179426_trimmed_hisat2_T2 M13v2.0.sorted.bam Coverage
ERR2179426_trimmed_hisat2_T2 M13v2.0.sorted.bam Junctions
ERR2179427_trimmed_hisat2_T2 M13v2.0.sorted.bam Coverage
ERR2179427_trimmed_hisat2_T2 M13v2.0.sorted.bam
ERR2179428_trimmed_hisat2_T2 M13v2.0.sorted.bam Coverage
ERR2179428_trimmed_hisat2_T2 M13v2.0.sorted.bam Junctions
ERR2179429_trimmed_hisat2_T2 M13v2.0.sorted.bam Coverage
ERR2179429_trimmed_hisat2_T2 M13v2.0.sorted.bam Junctions
ERR2179430_trimmed_hisat2_T2 M13v2.0.sorted.bam Coverage
ERR2179430_trimmed_hisat2_T2 M13v2.0.sorted.bam
ERR2179431_trimmed_hisat2_T2 M13v2.0.sorted.bam Coverage
ERR2179431_tr....0.sorted.bam
ERR2179432_trimmed_hisat2_T2 M13v2.0.sorted.bam Coverage
33 tracks loaded
NC_060926.1:130,893,7...
934M of 1,153M

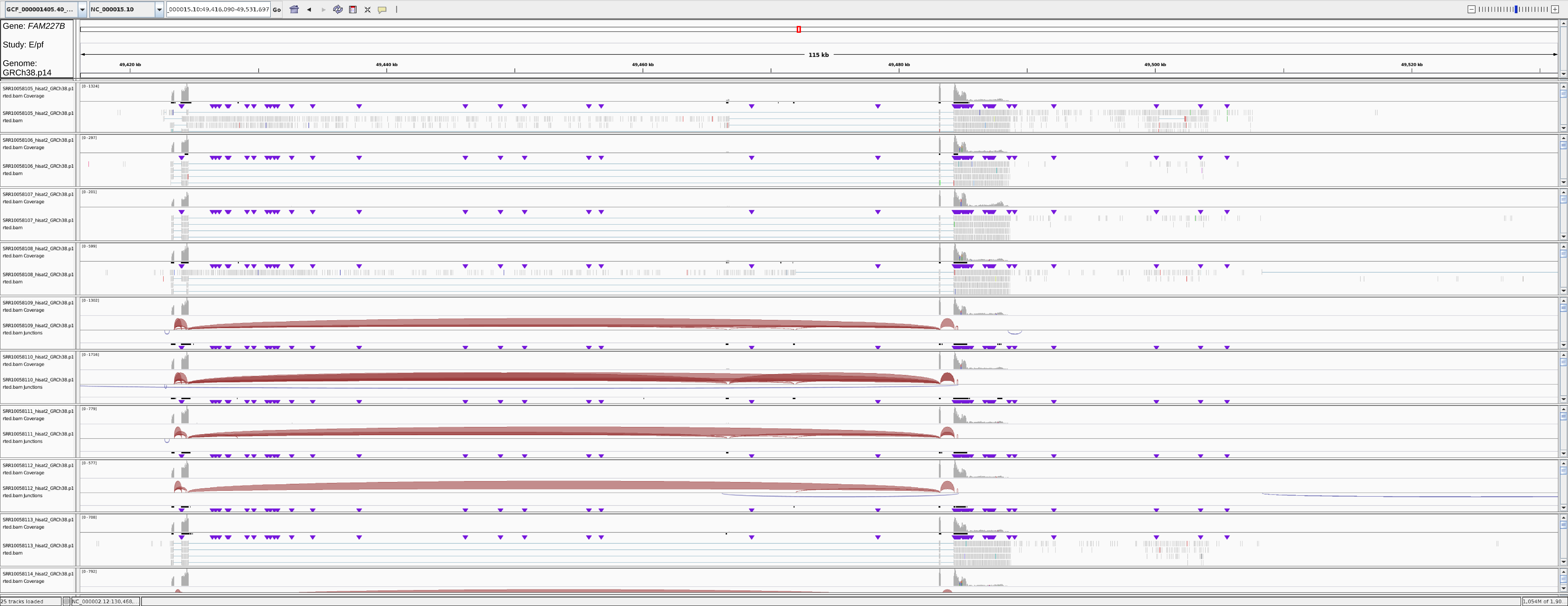

GCF_000001405.40_...
NC_000015.10
_000015.10:49,416,090-49,531,697
Go
Gene: FAM227B
Study: E/pf
Genome: GRCh38.p14
115 kb
49,420 kb
49,440 kb
49,460 kb
49,480 kb
49,500 kb
49,520 kb
SRR10058105_hisat2_GRCh38.p1 rted.bam Coverage
[0-1324]
SRR10058105_hisat2_GRCh38.p1 rted.bam
SRR10058106_hisat2_GRCh38.p1 rted.bam Coverage
[0-297]
SRR10058106_hisat2_GRCh38.p1 rted.bam
SRR10058107_hisat2_GRCh38.p1 rted.bam Coverage
[0-201]
SRR10058107_hisat2_GRCh38.p1 rted.bam
SRR10058108_hisat2_GRCh38.p1 rted.bam Coverage
[0-599]
SRR10058108_hisat2_GRCh38.p1 rted.bam
SRR10058109_hisat2_GRCh38.p1 rted.bam Coverage
[0-1302]
SRR10058109_hisat2_GRCh38.p1 rted.bam Junctions
SRR10058110_hisat2_GRCh38.p1 rted.bam Coverage
[0-1716]
SRR10058110_hisat2_GRCh38.p1 rted.bam Junctions
SRR10058111_hisat2_GRCh38.p1 rted.bam Coverage
[0-779]
SRR10058111_hisat2_GRCh38.p1 rted.bam Junctions
SRR10058112_hisat2_GRCh38.p1 rted.bam Coverage
[0-577]
SRR10058112_hisat2_GRCh38.p1 rted.bam Junctions
SRR10058113_hisat2_GRCh38.p1 rted.bam Coverage
[0-708]
SRR10058113_hisat2_GRCh38.p1 rted.bam
SRR10058114_hisat2_GRCh38.p1 rted.bam Coverage
[0-792]
25 tracks loaded
NC_000002.12:130,468,...

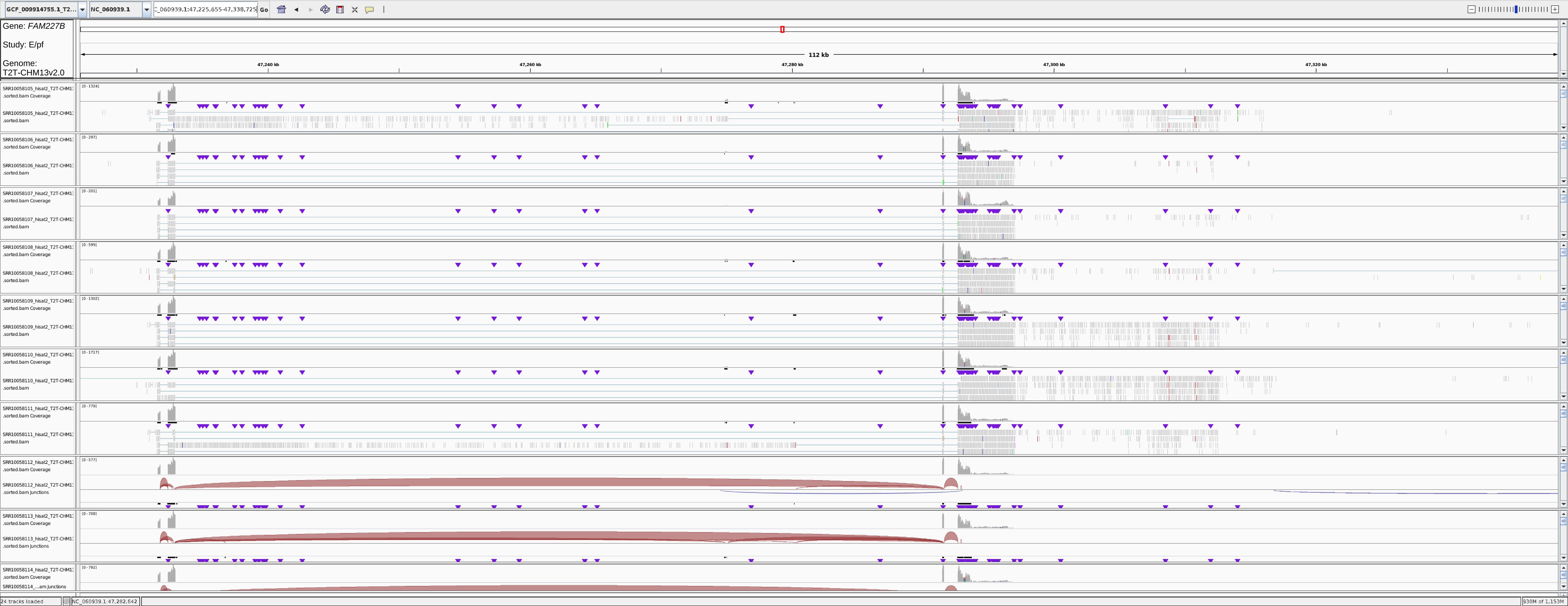

GCF_009914755.1_T2...
NC_060939.1
Go
Gene: FAM227B
Study: E/pf
Genome: T2T-CHM13v2.0
112 kb
47,240 kb
47,260 kb
47,280 kb
47,300 kb
47,320 kb
[0-1324]
[0-297]
[0-201]
[0-599]
[0-1302]
[0-1717]
[0-779]
[0-577]
[0-708]
[0-792]
24 tracks loaded
NC_060939.1:47,282,642
930M of 1,153M

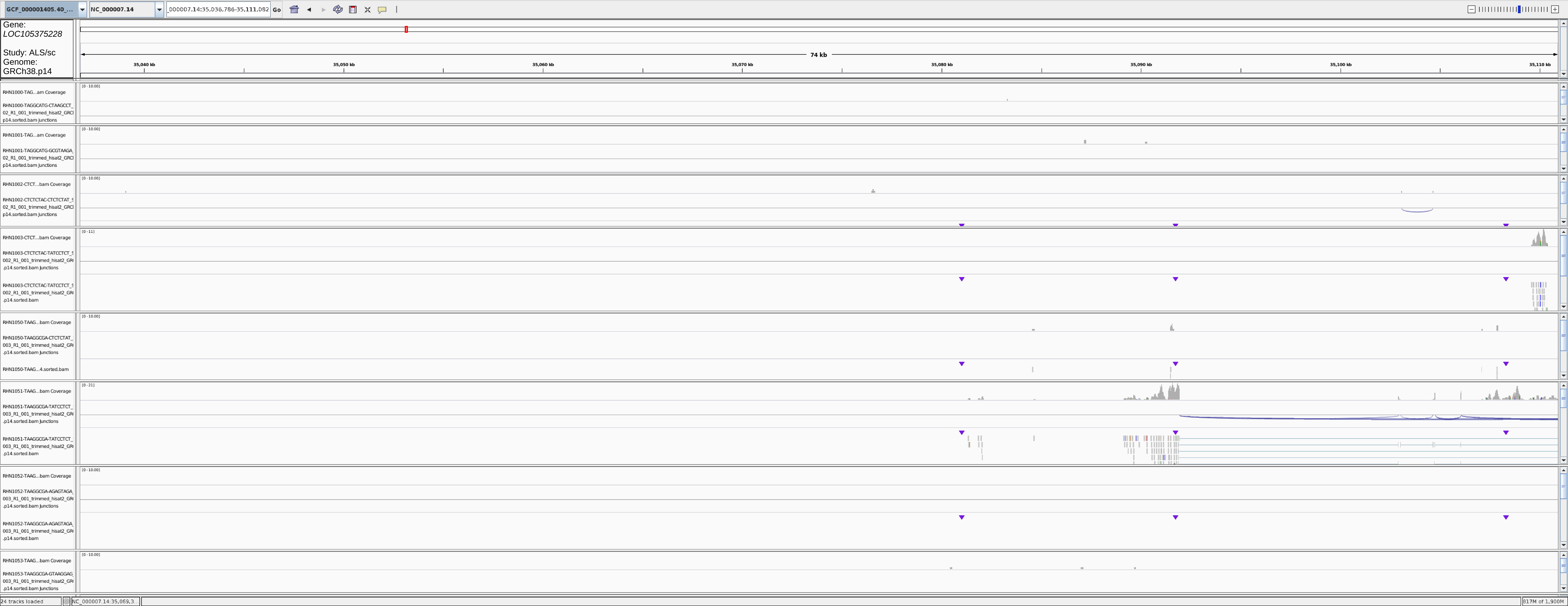
GCF_000001405.40_...
NC_000007.14
_000007.14:35,036,786-35,111,082
Go
Gene:
LOC105375228
Study: ALS/sc
Genome:
GRCh38.p14
74 kb
35,040 kb
35,050 kb
35,060 kb
35,070 kb
35,080 kb
35,090 kb
35,100 kb
35,110 kb
RHN1000-TAG...am Coverage
RHN1000-TAGGCATG-CTAAGCCT_
02_R1_001_trimmed_hisat2_GRC
p14.sorted.bam Junctions
RHN1001-TAG...am Coverage
RHN1001-TAGGCATG-GCGTAAGA_
02_R1_001_trimmed_hisat2_GRC
p14.sorted.bam Junctions
RHN1002-CTCT...bam Coverage
RHN1002-CTCTCTAC-CTCTCTAT_
02_R1_001_trimmed_hisat2_GRC
p14.sorted.bam Junctions
RHN1003-CTCT...bam Coverage
RHN1003-CTCTCTAC-TATCCTCT_
002_R1_001_trimmed_hisat2_GR
.p14.sorted.bam Junctions
RHN1003-CTCTCTAC-TATCCTCT_
002_R1_001_trimmed_hisat2_GR
.p14.sorted.bam
RHN1050-TAAG...bam Coverage
RHN1050-TAAGGCGA-CTCTCTAT_
003_R1_001_trimmed_hisat2_GR
.p14.sorted.bam Junctions
RHN1050-TAAG...4.sorted.bam
RHN1051-TAAG...bam Coverage
RHN1051-TAAGGCGA-TATCCTCT_
003_R1_001_trimmed_hisat2_GR
.p14.sorted.bam Junctions
RHN1051-TAAGGCGA-TATCCTCT_
003_R1_001_trimmed_hisat2_GR
.p14.sorted.bam
RHN1052-TAAG...bam Coverage
RHN1052-TAAGGCGA-AGAGTAGA_
003_R1_001_trimmed_hisat2_GR
.p14.sorted.bam Junctions
RHN1052-TAAGGCGA-AGAGTAGA_
003_R1_001_trimmed_hisat2_GR
.p14.sorted.bam
RHN1053-TAAG...bam Coverage
RHN1053-TAAGGCGA-GTAAGGAG_
003_R1_001_trimmed_hisat2_GR
.p14.sorted.bam Junctions
24 tracks loaded
NC_000007.14:35,069,3...
817M of 1,900M

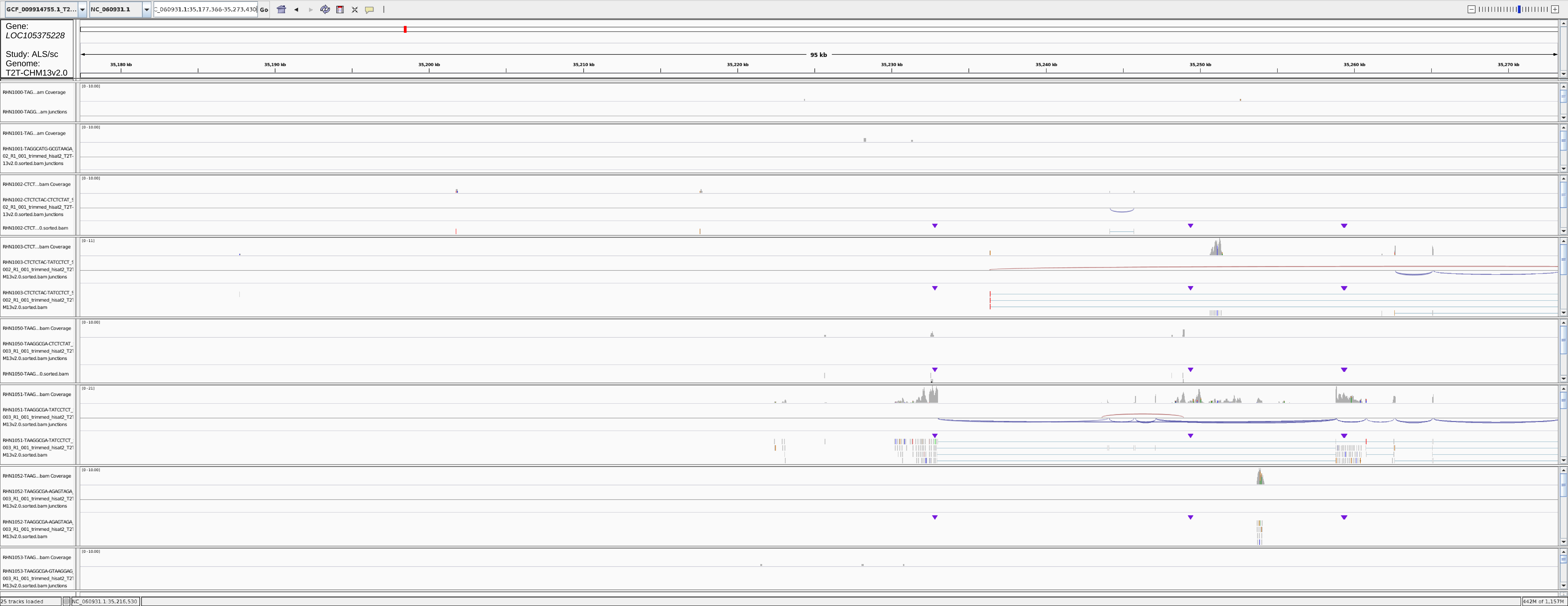
GCF_009914755.1_T2...
NC_060931.1
Go
Gene:
LOC105375228
Study: ALS/sc
Genome:
T2T-CHM13v2.0
95 kb
35,180 kb
35,190 kb
35,200 kb
35,210 kb
35,220 kb
35,230 kb
35,240 kb
35,250 kb
35,260 kb
35,270 kb
RHN1000-TAG...am Coverage
RHN1000-TAGG...am Junctions
RHN1001-TAG...am Coverage
RHN1001-TAGGCATG-GCGTAAGA
02_R1_001_trimmed_hisat2_T2T-
13v2.0.sorted.bam Junctions
RHN1002-CTCT...bam Coverage
RHN1002-CTCTCTAC-CTCTCTAT_
02_R1_001_trimmed_hisat2_T2T-
13v2.0.sorted.bam Junctions
RHN1002-CTCT...0.sorted.bam
RHN1003-CTCT...bam Coverage
RHN1003-CTCTCTAC-TATCCTCT_
002_R1_001_trimmed_hisat2_T2T
M13v2.0.sorted.bam Junctions
RHN1003-CTCTCTAC-TATCCTCT_
002_R1_001_trimmed_hisat2_T2T
M13v2.0.sorted.bam
RHN1050-TAAG...bam Coverage
RHN1050-TAAGGCGA-CTCTCTAT_
003_R1_001_trimmed_hisat2_T2T
M13v2.0.sorted.bam Junctions
RHN1050-TAAG...0.sorted.bam
RHN1051-TAAG...bam Coverage
RHN1051-TAAGGCGA-TATCCTCT_
003_R1_001_trimmed_hisat2_T2T
M13v2.0.sorted.bam Junctions
RHN1051-TAAGGCGA-TATCCTCT_
003_R1_001_trimmed_hisat2_T2T
M13v2.0.sorted.bam
RHN1052-TAAG...bam Coverage
RHN1052-TAAGGCGA-AGAGTAGA_
003_R1_001_trimmed_hisat2_T2T
M13v2.0.sorted.bam Junctions
RHN1052-TAAGGCGA-AGAGTAGA_
003_R1_001_trimmed_hisat2_T2T
M13v2.0.sorted.bam
RHN1053-TAAG...bam Coverage
RHN1053-TAAGGCGA-GTAAGGAG_
003_R1_001_trimmed_hisat2_T2T
M13v2.0.sorted.bam Junctions
25 tracks loaded
NC_060931.1:35,216,530
442M of 1,157M
