# Supplementary material for: Transcriptome driven discovery of novel candidate genes for human neurological disorders in the telomer-to-telomer genome assembly era
Source: Hum Genomics. 2023 Oct 23;17:94. doi: 10.1186/s40246-023-00543-y (PMC10594789; doi:10.1186/s40246-023-00543-y)
Supplement: Supplementary file 7 — Additional file 7. Volcano plots of differential expression analysis with the T2T-CHM13v2.0 assembly, including genes, which were only discovered using the GRCh13.p14 assembly. [file 40246_2023_543_MOESM7_ESM.pdf]

AD/n

Additional File S7: Volcano plots of differential expression analysis with the T2T-CHM13v2.0 assembly, including genes, which were only discovered using the GRCh13.p14 assembly.

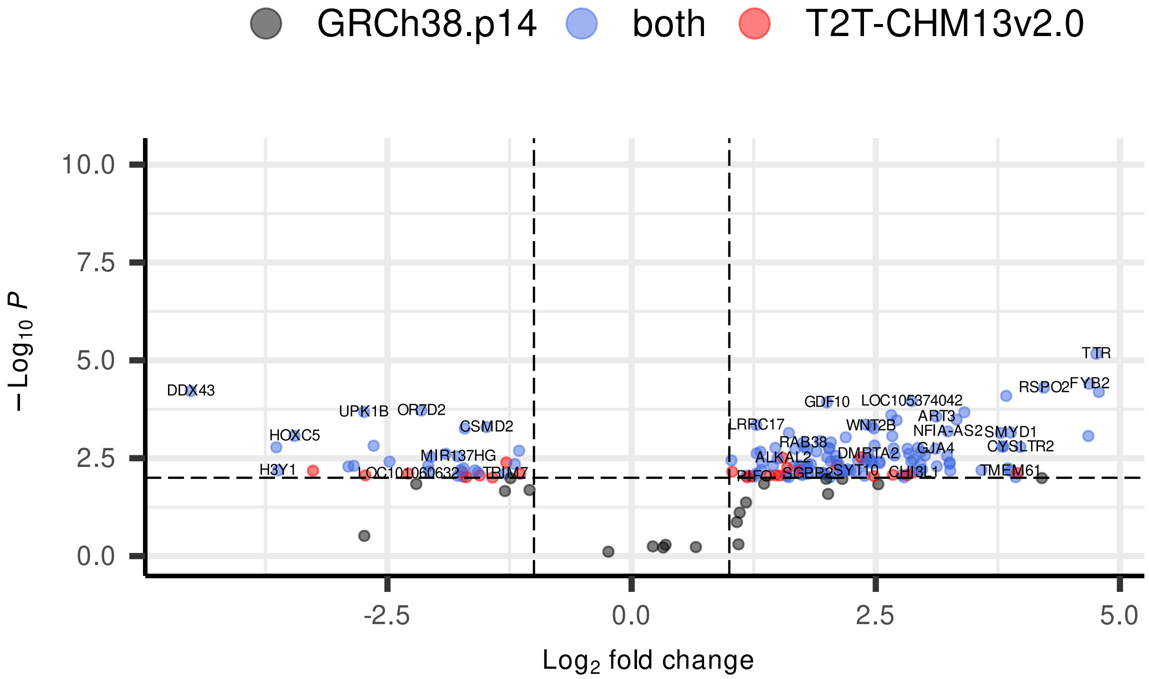

total = 182 variables

N(transcripts) p.adj<0.01 / abs. Log2 FC <1 = 5

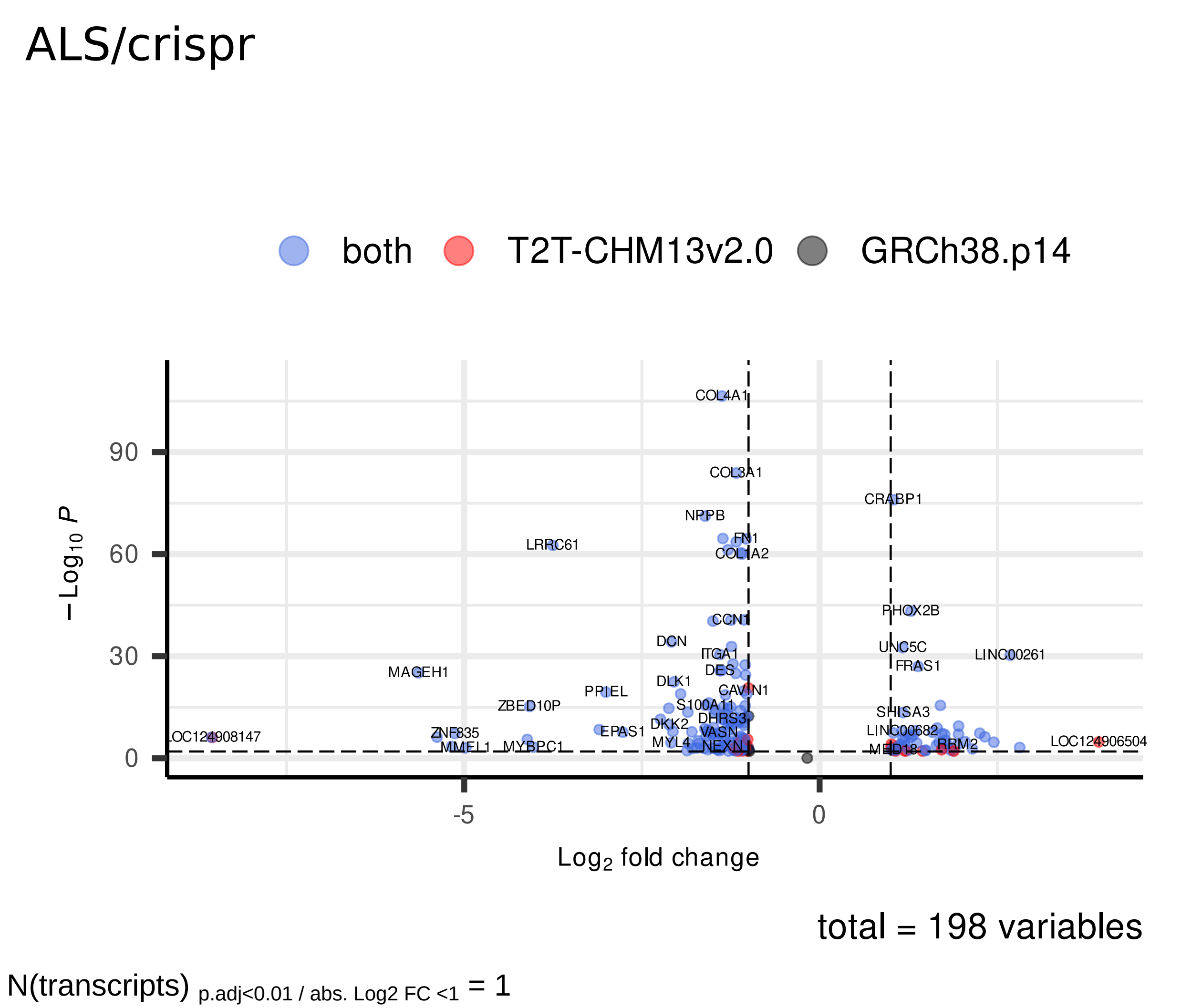
ALS/crispr
both
T2T-CHM13v2.0
GRCh38.p14
COL4A1
COL3A1
CRABP1
NPPB
FN1
LRRC61
COL1A2
PHOX2B
CCN1
DCN
UNC5C
LINC00261
ITGA1
FRAS1
DES
MAGEH1
DLK1
PPIEL
CAVIN1
S100A11
ZBED10P
SHISA3
DHRS3
DKK2
LINC00682
EPAS1
VASN
ZNF835
LOC124908147
LOC124906504
MYL4
RRM2
MMEL1
MYBPC1
NEXN
MED18
90
60
30
0
-5
0
$-Log_{10}$ P
Log_2 fold change
total = 198 variables
N(transcripts) $_{p.adj<0.01 / abs. Log2 FC <1}$ = 1

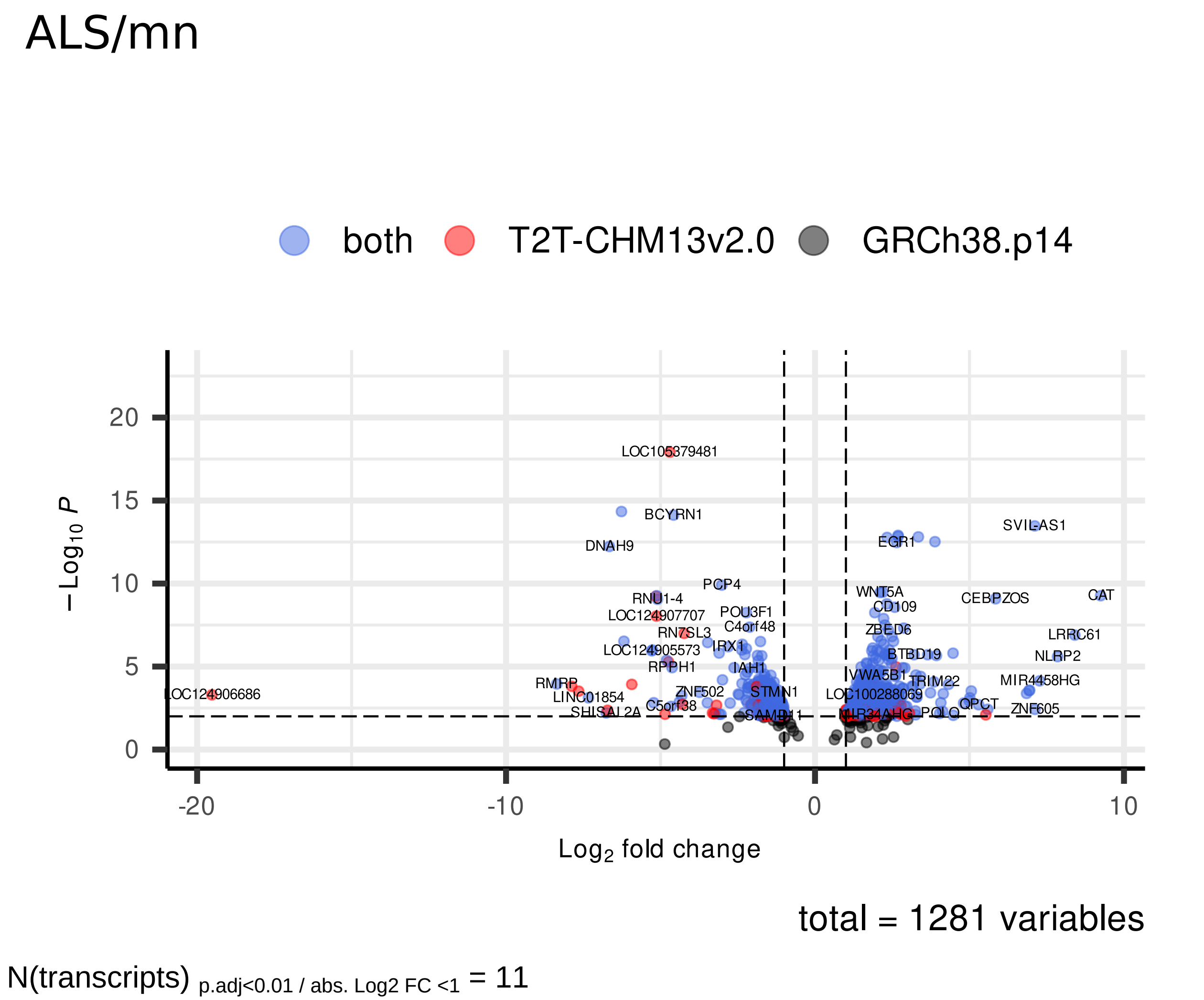
ALS/mn
both
T2T-CHM13v2.0
GRCh38.p14
−Log10 P
Log2 fold change
20
15
10
5
0
-20
-10
0
10
LOC105379481
BCYRN1
DNAH9
SVIL-AS1
EGR1
PCP4
RNU1-4
LOC124907707
POU3F1
C4orf48
RN7SL3
LOC124905573
IRX1
RPPH1
IAH1
RMRP
LINC01854
SHISAL2A
C5orf38
ZNF502
STMN1
SAMD11
LOC124906686
WNT5A
CD109
CEBPZOS
CAT
ZBED6
LRRC61
BTBD19
NLRP2
VWA5B1
TRIM22
MIR4458HG
LOC100288069
NLR34AHG
POLQ
QPCT
ZNF605
total = 1281 variables
N(transcripts) p.adj<0.01 / abs. Log2 FC <1 = 11

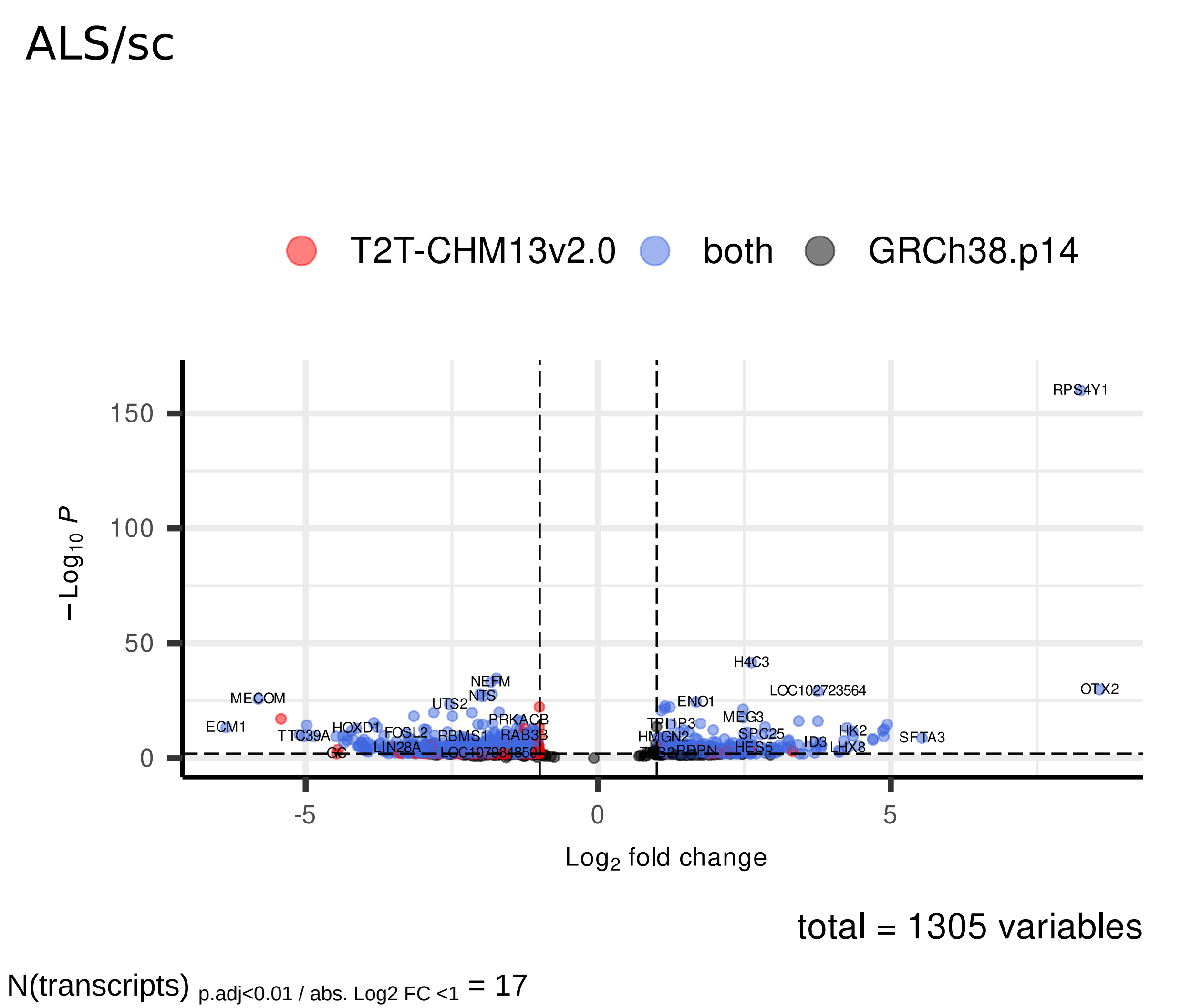

ALS/sc
T2T-CHM13v2.0
both
GRCh38.p14
RPS4Y1
H4C3
LOC102723564
OTX2
NEFM
NTS
UTS2
MECOM
ENO1
ECM1
TTC39A
HOXD1
FOSL2
PRKACB
TPI1P3
MEG3
RBMS1
RAB3B
HMGN2
SPC25
HK2
SFTA3
ID3
LHX8
HES5
PDPN
LIN28A
GC
150
100
50
0
$-\text{Log}_{10}\ P$
-5
0
5
Log_2 fold change
total = 1305 variables
N(transcripts) p.adj<0.01 / abs. Log2 FC <1 = 17

ASD/nsc

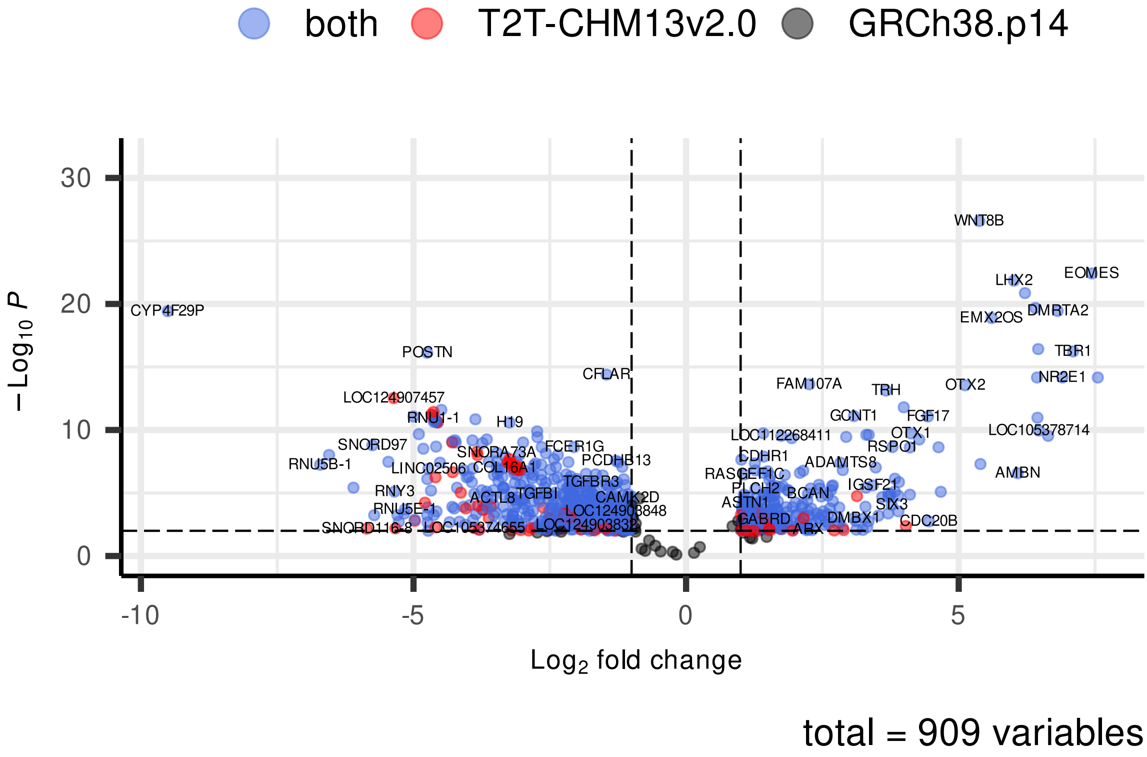

N(transcripts) $_{\text{p.adj<0.01 / abs. Log2 FC <1}}$ = 13

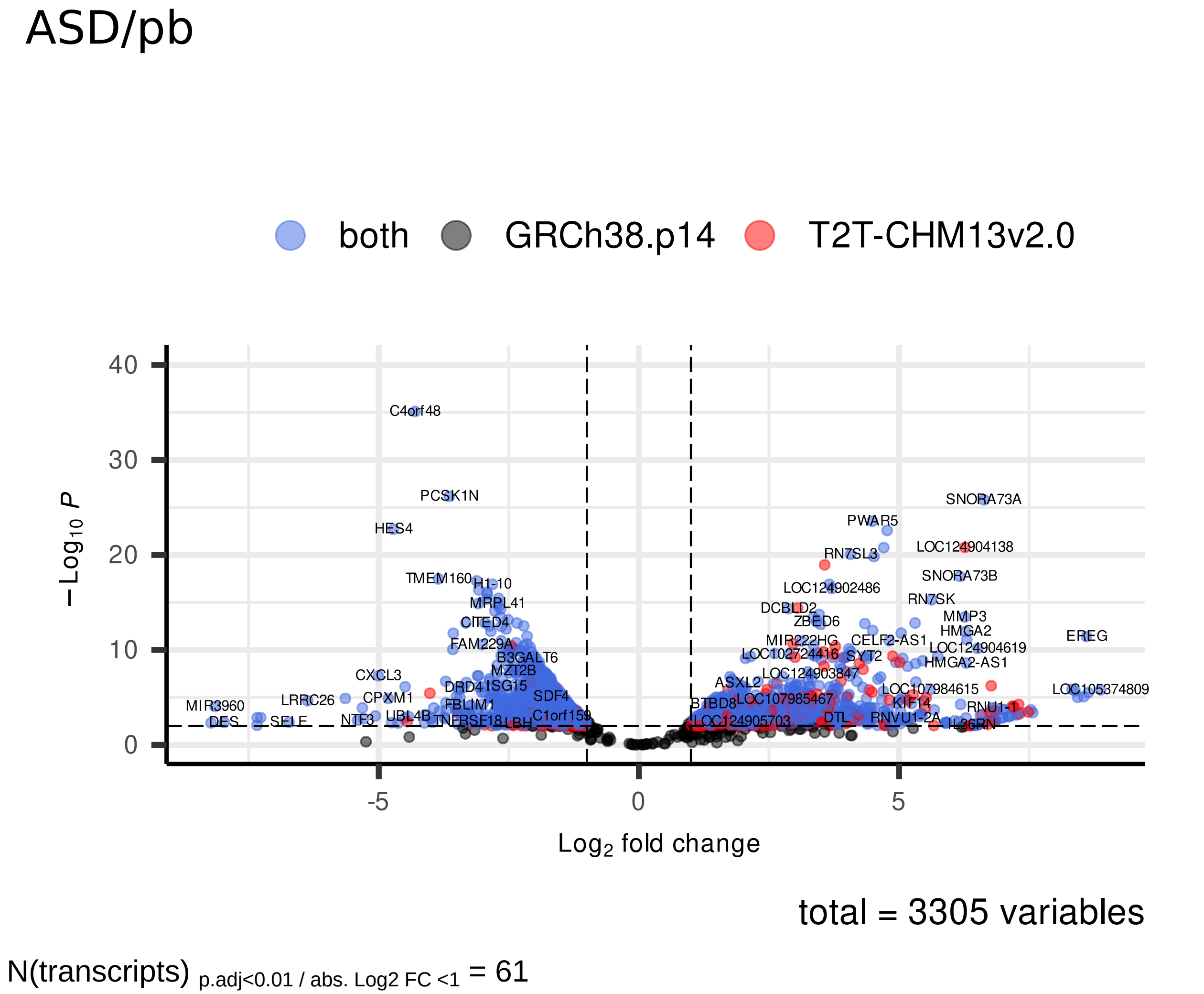

ASD/pb
both
GRCh38.p14
T2T-CHM13v2.0
−Log10 P
Log2 fold change
40
30
20
10
0
-5
0
5
C4orf48
PCSK1N
HES4
TMEM160
H1-10
MRPL41
CITED4
FAM229A
B3GALT6
MZT2B
CXCL3
DRD4
ISG15
SDF4
LRRC26
CPXM1
FBLIM1
MIR3960
DES
SELE
NTF3
UBL4B
TNFRSF18
LBH
C1orf159
SNORA73A
PWAR5
LOC124904138
RN7SL3
SNORA73B
LOC124902486
RN7SK
DCBLD2
ZBED6
MMP3
HMGA2
EREG
MIR222HG
CELF2-AS1
LOC124904619
LOC102724416
SYT2
HMGA2-AS1
LOC124903847
ASXL2
LOC107984615
LOC105374809
BTBD8
LOC107985467
KIF14
RNU1-1
LOC124905703
DTL
RNVU1-2A
IL36RN
total = 3305 variables
N(transcripts) p.adj<0.01 / abs. Log2 FC <1 = 61

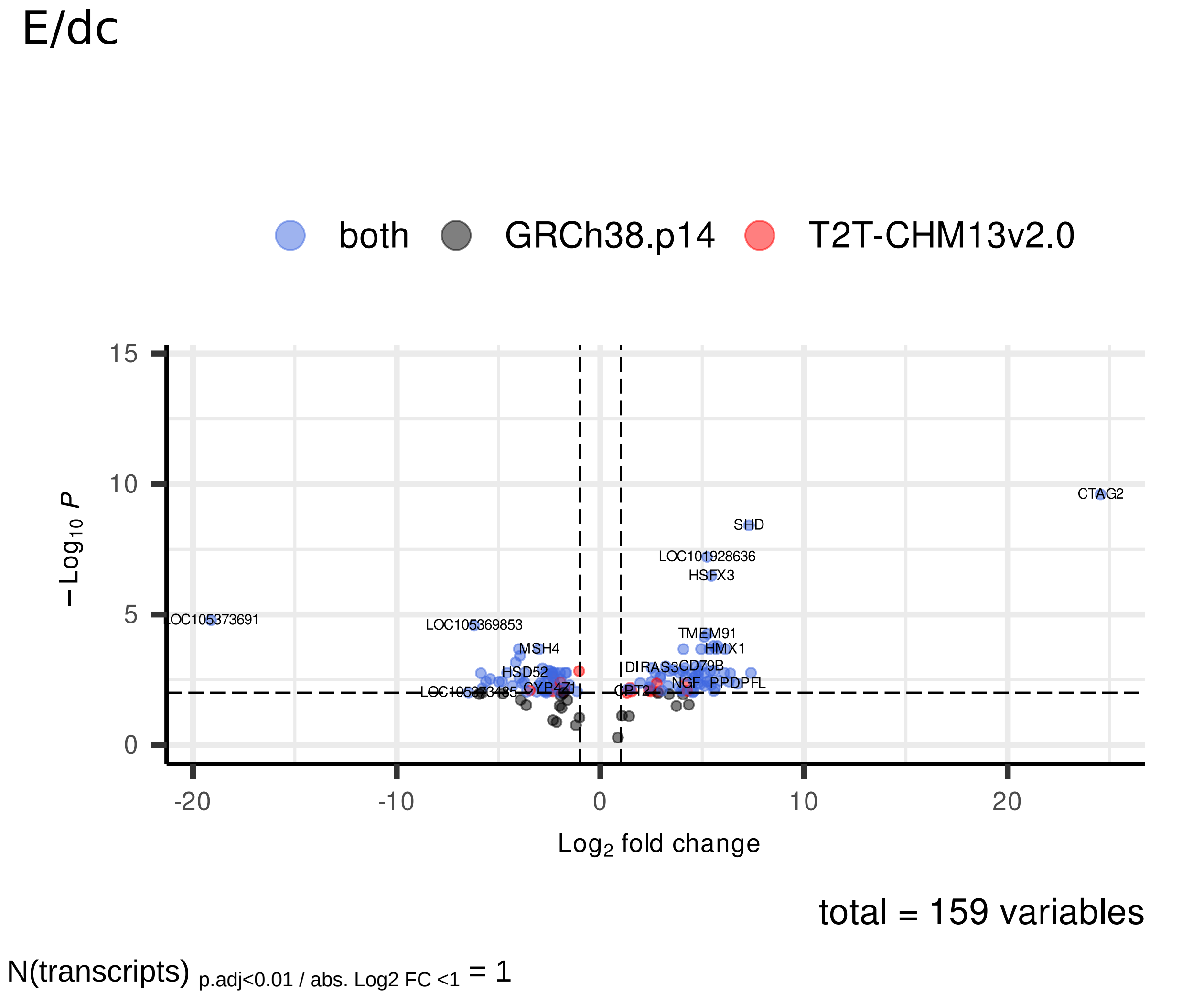

E/dc
both
GRCh38.p14
T2T-CHM13v2.0
15
10
5
0
$-\text{Log}_{10}\,P$
-20
-10
0
10
20
Log_2 fold change
CTAG2
SHD
LOC101928636
HSFX3
LOC105373691
LOC105369853
TMEM91
MSH4
HMX1
DIRAS3
CD79B
HSD52
NGF
PPDPFL
CYP4Z1
CPT2
total = 159 variables
N(transcripts) $_{\text{p.adj<0.01 / abs. Log2 FC <1}}$ = 1

E/na

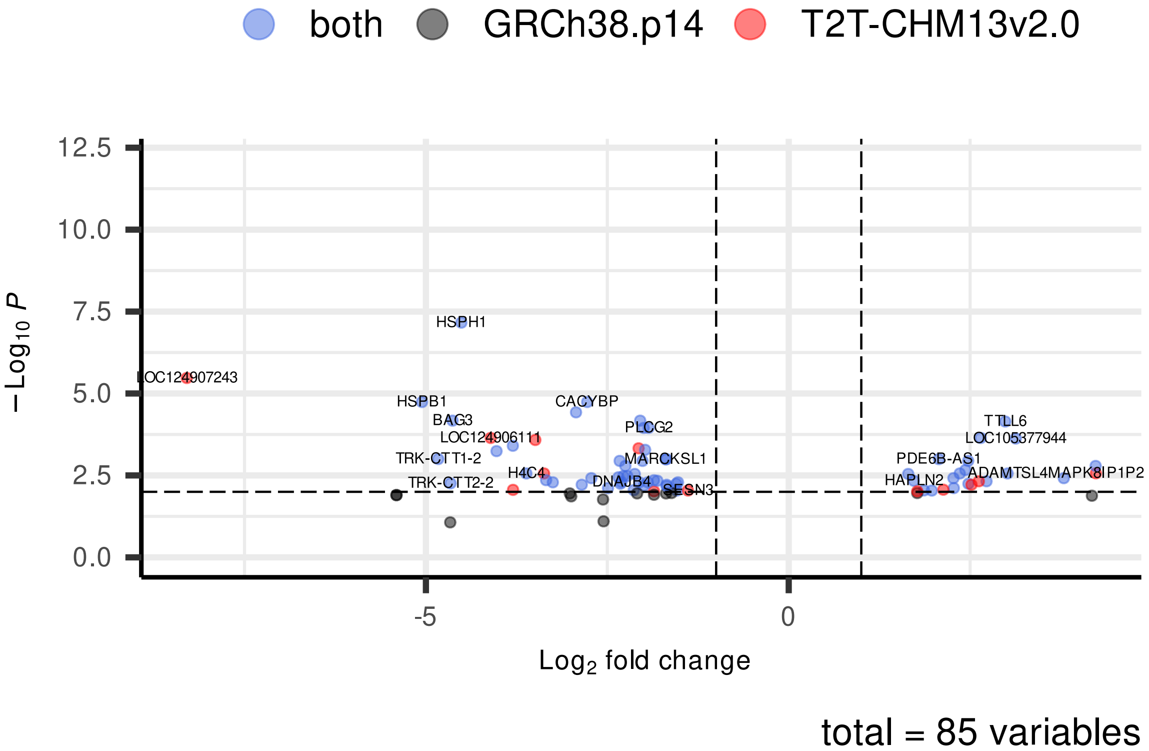

N(transcripts) $_{\text{p.adj<0.01 / abs. Log2 FC <1}}$ = 0

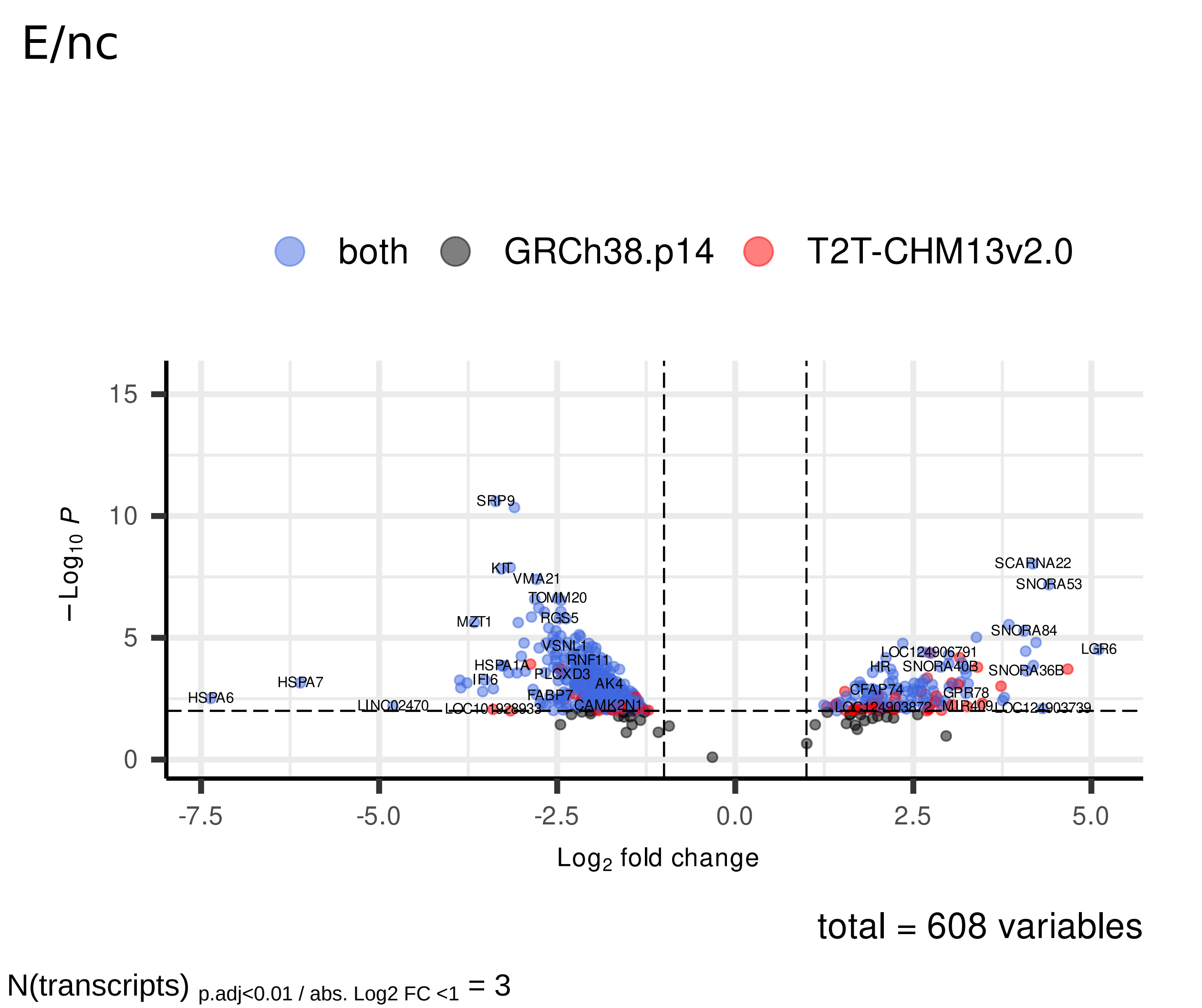
E/nc
both
GRCh38.p14
T2T-CHM13v2.0
−Log10 P
Log2 fold change
SRP9
KIT
VMA21
TOMM20
MZT1
RGS5
VSNL1
RNF11
HSPA1A
PLCXD3
IFI6
AK4
FABP7
CAMK2N1
HSPA7
HSPA6
LINC02470
LOC101928933
SCARNA22
SNORA53
SNORA84
LGR6
LOC124906791
HR
SNORA40B
SNORA36B
CFAP74
GPR78
LOC124903872
MIR409
LOC124903739
total = 608 variables
N(transcripts) p.adj<0.01 / abs. Log2 FC <1 = 3

E/nopc

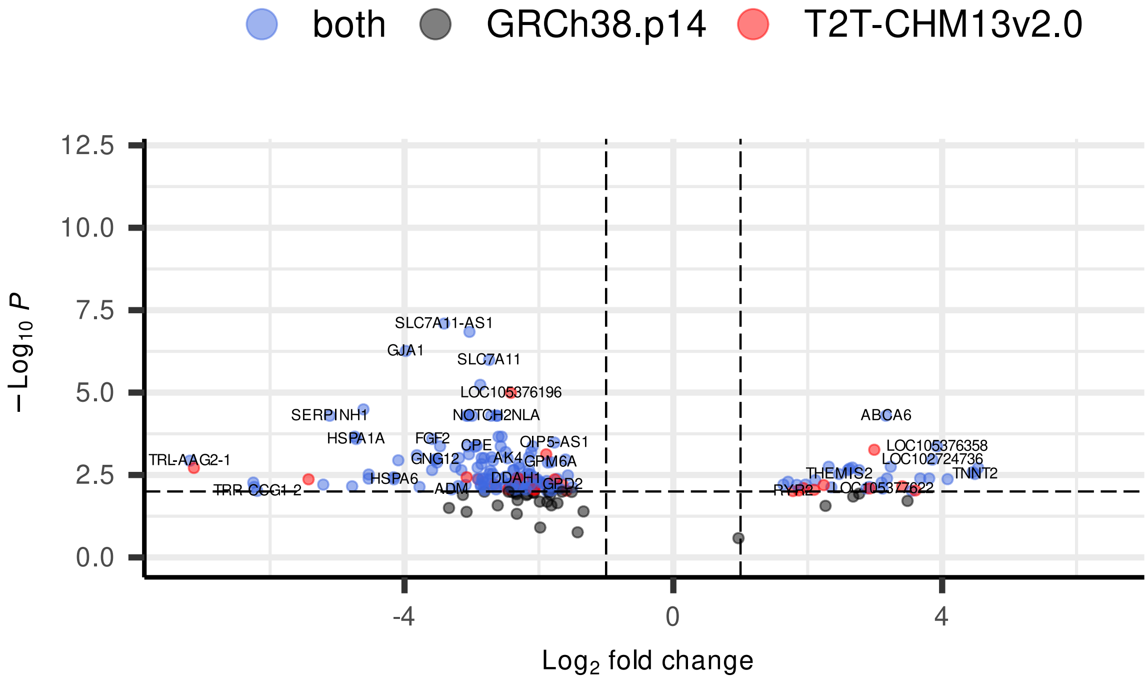

total = 221 variables

N(transcripts) $_{\text{p.adj<0.01 / abs. Log2 FC <1}}$ = 1

E/pf

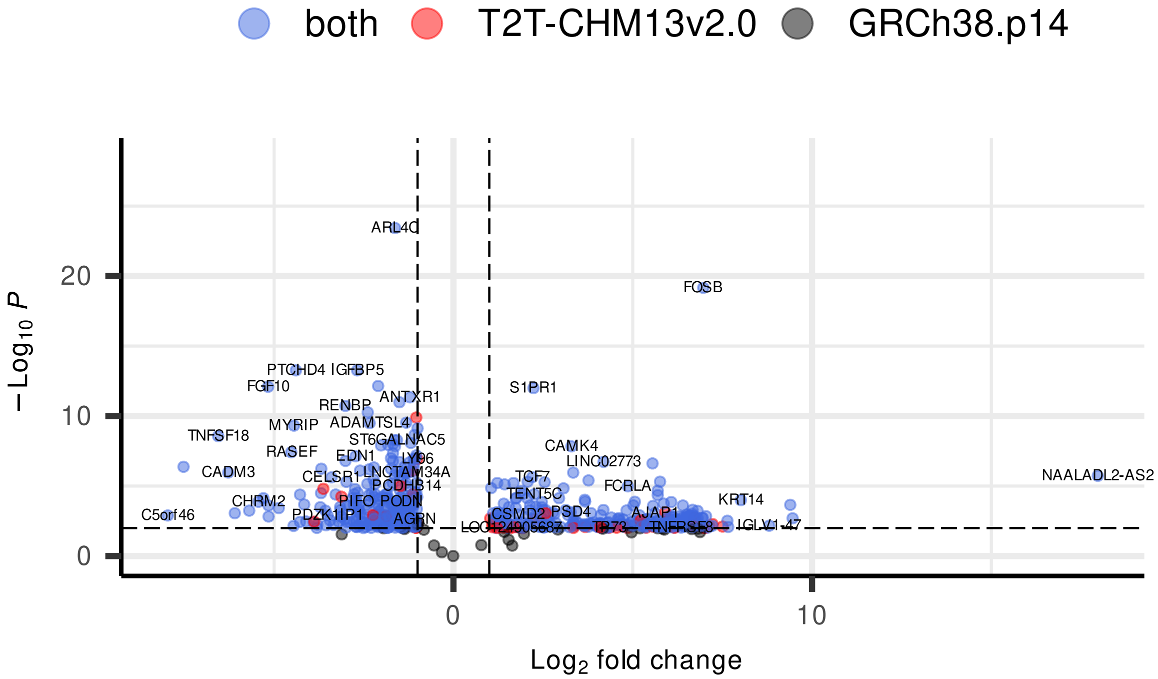

total = 616 variables

N(transcripts) $_{\text{p.adj<0.01 / abs. Log2 FC <1}}$ = 5

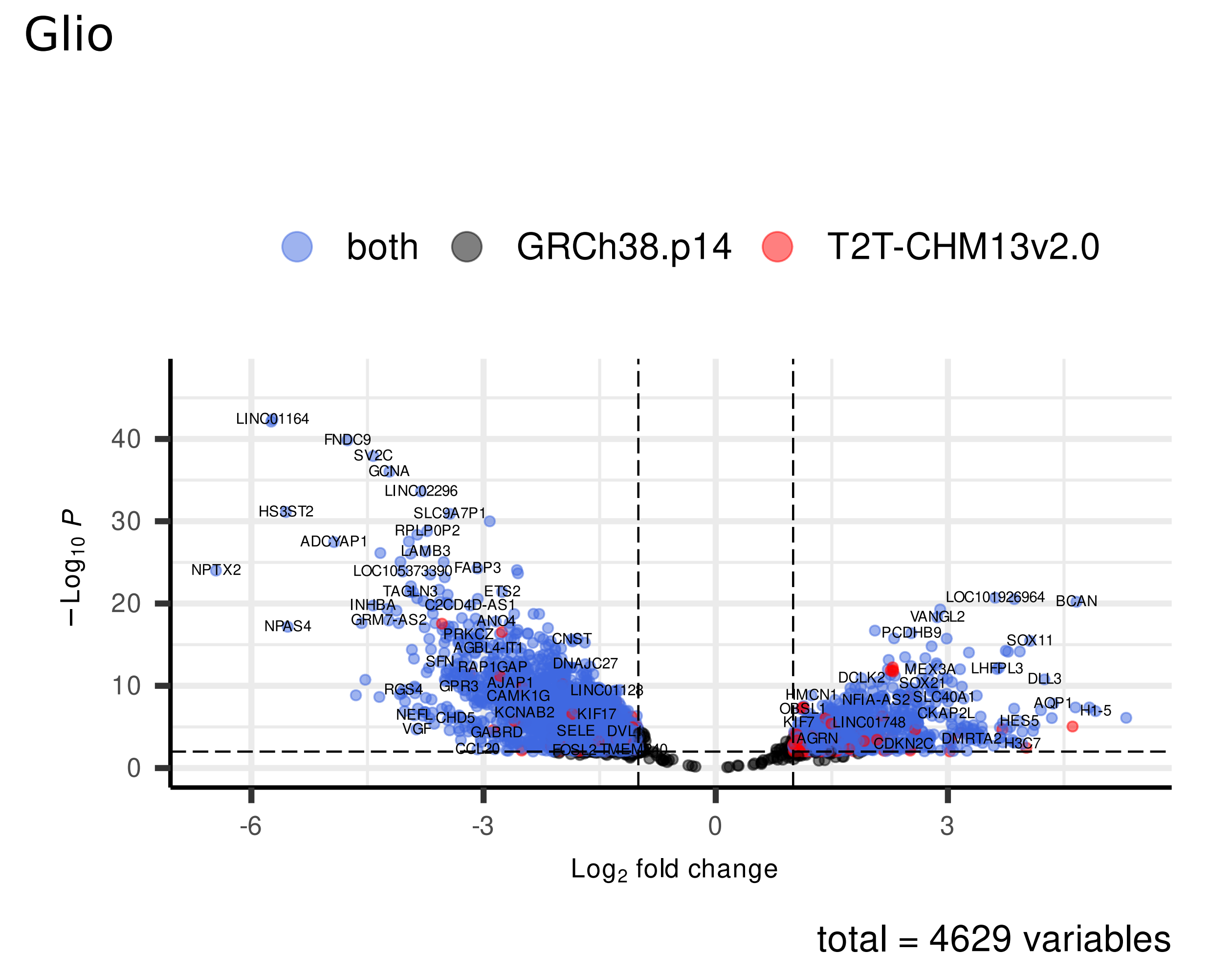

N(transcripts) $_{\text{p.adj<0.01 / abs. Log2 FC <1}}$ = 48

Gliob/ec

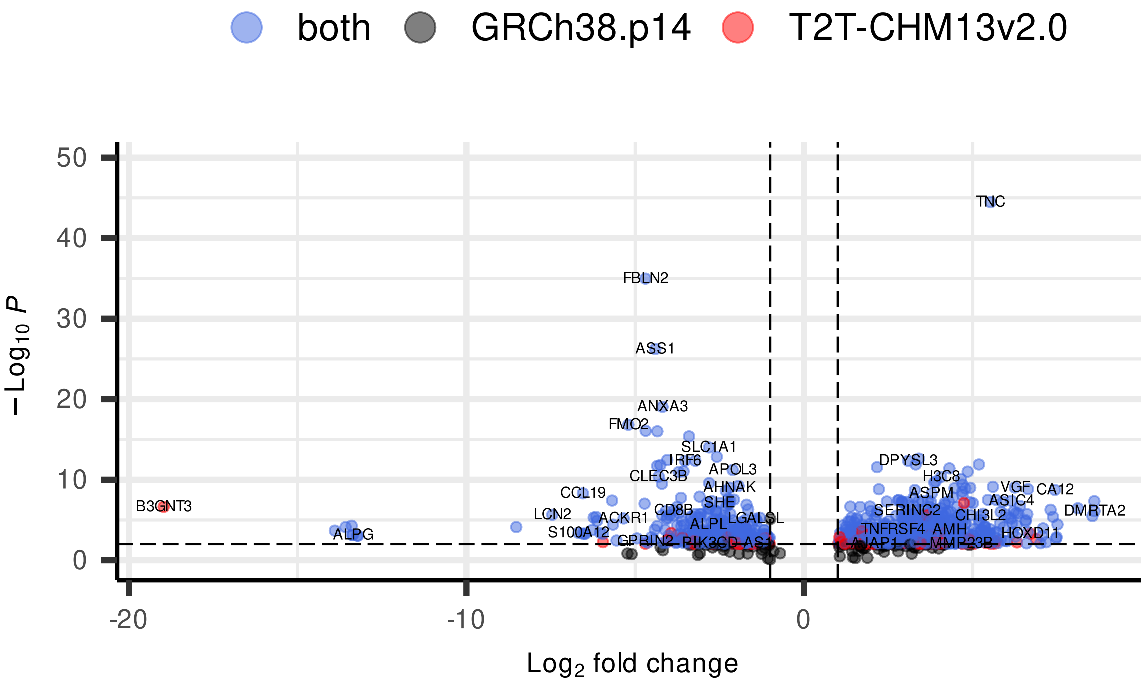

total = 1144 variables

N(transcripts) $_{\text{p.adj<0.01 / abs. Log2 FC <1}}$ = 3

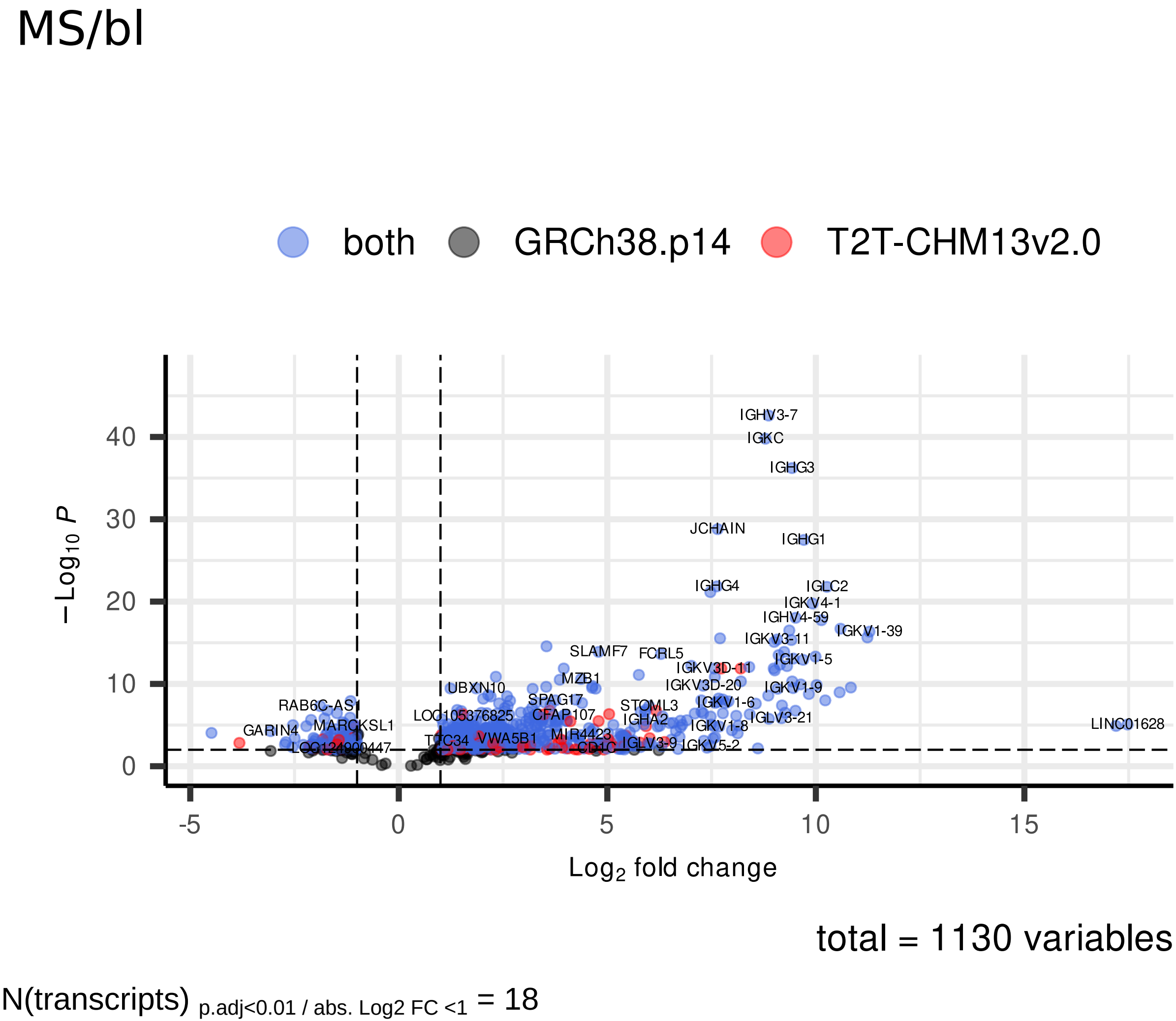

MS/bl
both
GRCh38.p14
T2T-CHM13v2.0
−Log10 P
40
30
20
10
0
-5
0
5
10
15
Log2 fold change
IGHV3-7
IGKC
IGHG3
JCHAIN
IGHG1
IGHG4
IGLC2
IGKV4-1
IGHV4-59
IGKV1-39
IGKV3-11
SLAMF7
FCRL5
IGKV1-5
IGKV3D-11
MZB1
UBXN10
IGKV3D-20
IGKV1-9
SPAG17
IGKV1-6
RAB6C-AS1
STOML3
LOC105376825
CFAP107
IGHA2
IGLV3-21
MARCKSL1
IGKV1-8
LINC01628
GARIN4
TTC34
VWA5B1
MIR4423
IGLV3-9
IGKV5-2
CD1C
LOC124900447
total = 1130 variables
N(transcripts) p.adj<0.01 / abs. Log2 FC <1 = 18

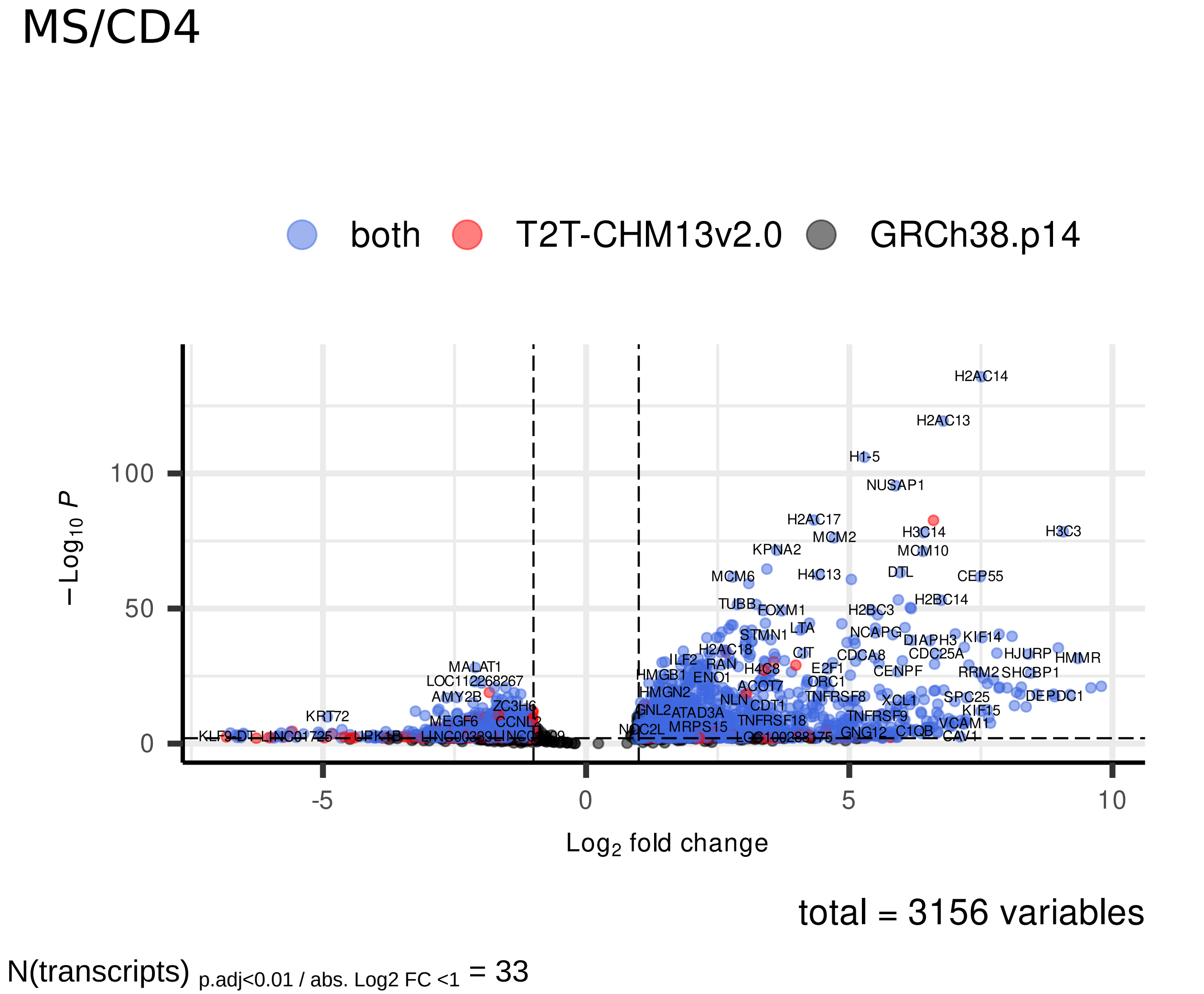
MS/CD4
both
T2T-CHM13v2.0
GRCh38.p14
H2AC14
H2AC13
H1-5
NUSAP1
H2AC17
H3C14
H3C3
MCM2
KPNA2
MCM10
MCM6
H4C13
DTL
CEP55
TUBB
FOXM1
H2BC3
H2BC14
LTA
STMN1
NCAPG
DIAPH3
KIF14
H2AC18
CIT
CDCA8
CDC25A
HJURP
HMMR
ILF2
RAN
H4C8
E2F1
CENPF
RRM2
SHCBP1
HMGB1
ENO1
ORC1
ACOT7
HMGN2
NLN
CDT1
TNFRSF8
XCL1
SPC25
DEPDC1
GNL2
ATAD3A
TNFRSF9
KIF15
TNFRSF18
VCAM1
MRPS15
NOC2L
GNG12
C1QB
CAV1
MALAT1
LOC112268267
AMY2B
ZC3H6
KRT72
MEGF6
CCNL2
KLF9-DT
LINC01725
UPK1B
LINC00339
100
50
0
-5
0
5
10
$-Log_{10}$ P
Log_2 fold change
total = 3156 variables
N(transcripts) p.adj<0.01 / abs. Log2 FC <1 = 33

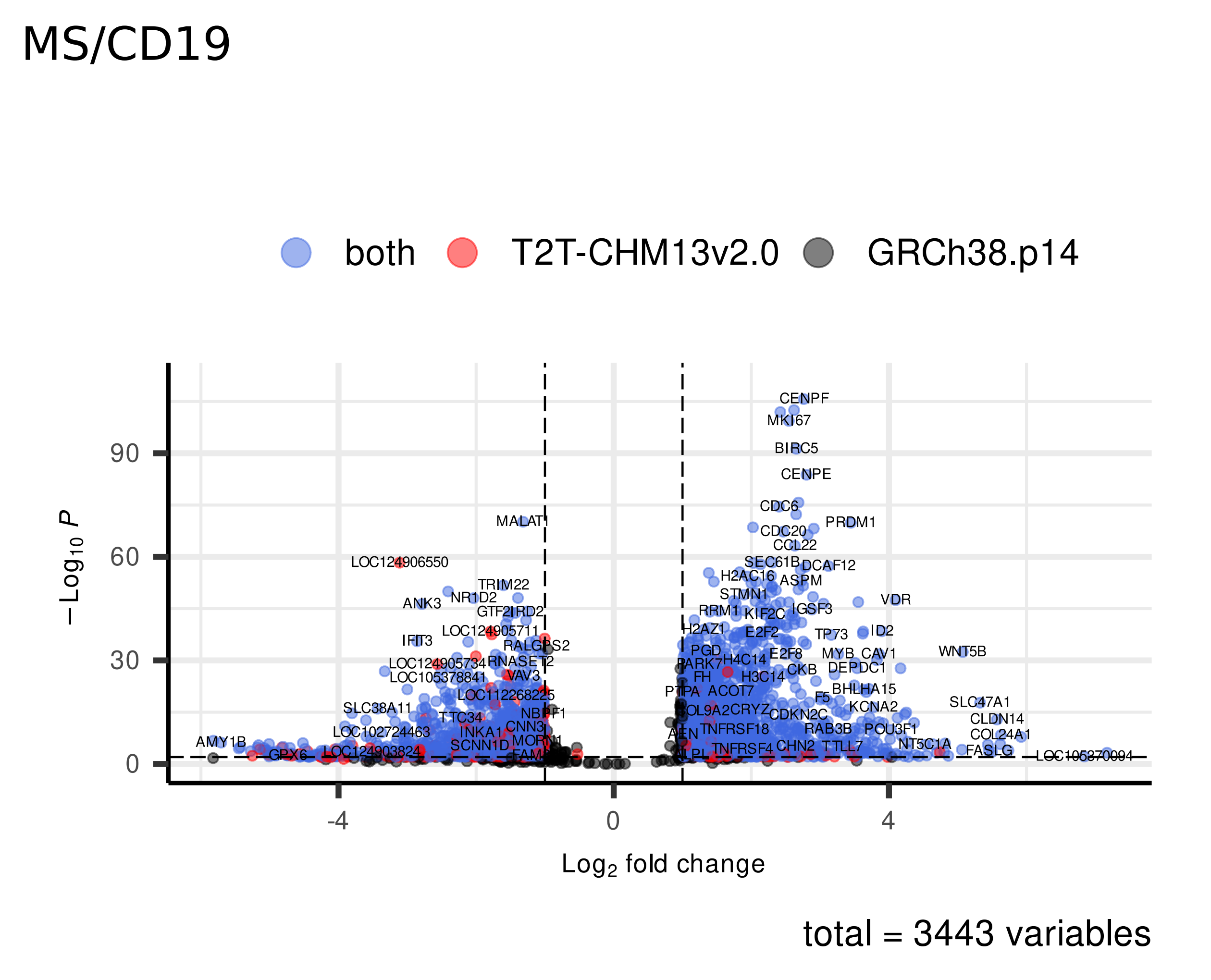

N(transcripts) $_{\text{p.adj<0.01 / abs. Log2 FC <1}}$ = 36

mtDNA/m.3243

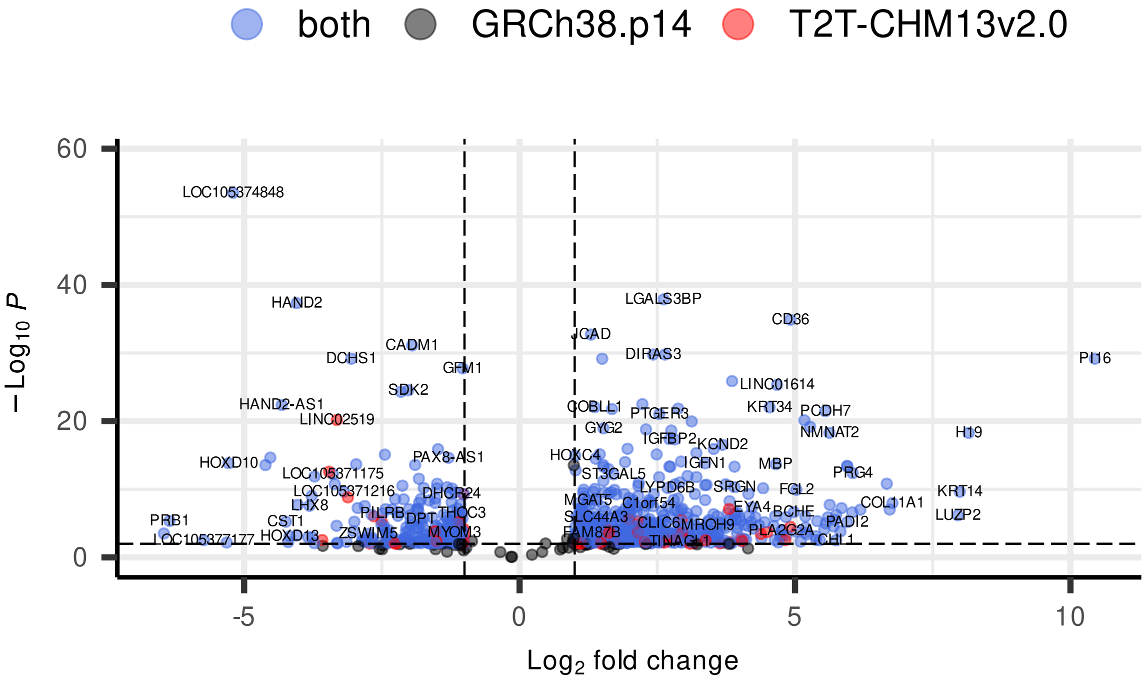

total = 876 variables

N(transcripts) $_{\text{p.adj<0.01 / abs. Log2 FC <1}}$ = 9
