# Supplementary material for: Transcriptome driven discovery of novel candidate genes for human neurological disorders in the telomer-to-telomer genome assembly era
Source: Hum Genomics. 2023 Oct 23;17:94. doi: 10.1186/s40246-023-00543-y (PMC10594789; doi:10.1186/s40246-023-00543-y)

Additional File S9: Dimer (A), trimer (B), and tetramer (C) structure prediction of the LOC124906582 protein with Alphafold.

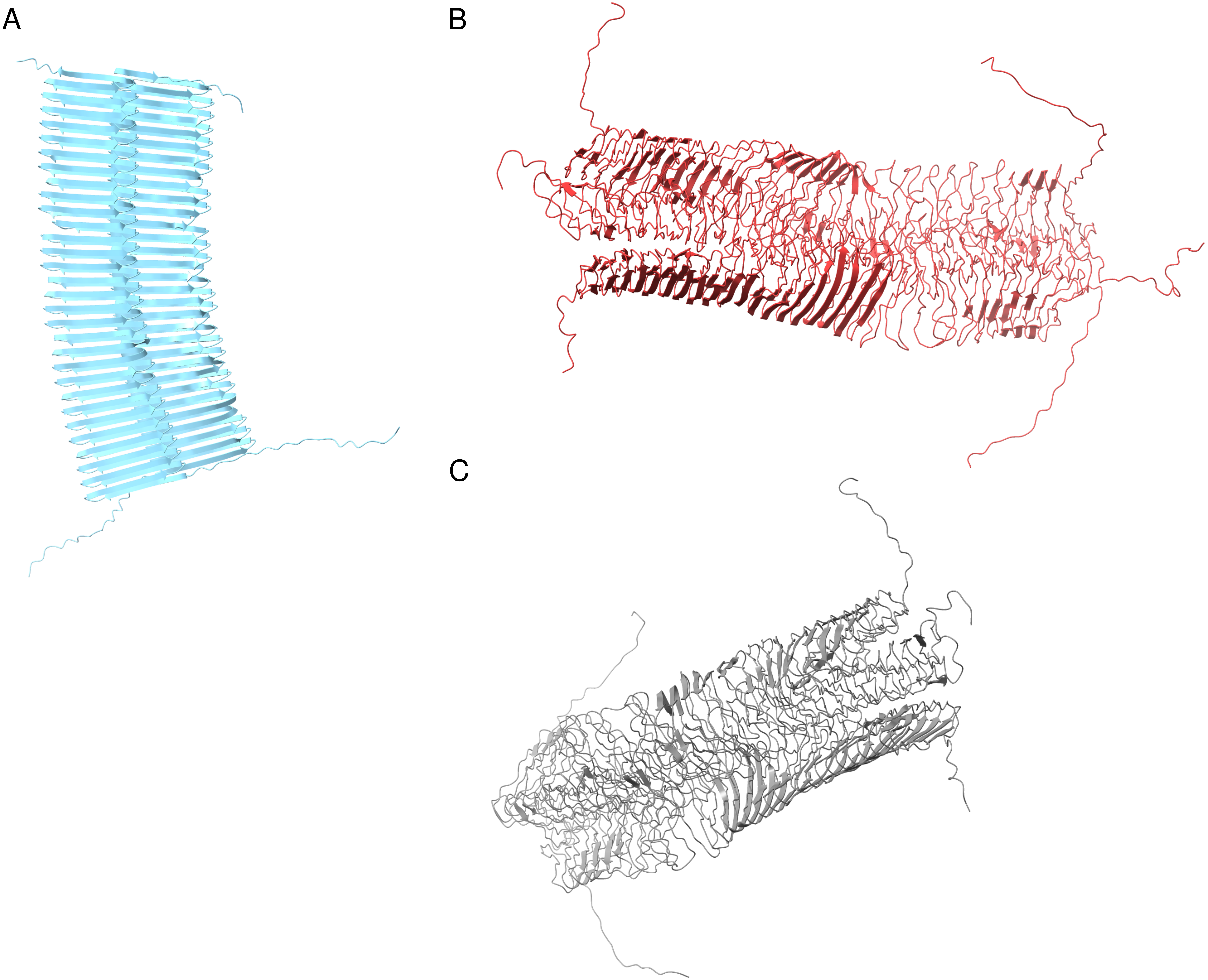

Supplement: Supplementary file 9 — Additional file 9. Dimer (A), trimer (B), and tetramer (C) structure prediction of the LOC124906582 protein with Alphafold. [file 40246_2023_543_MOESM9_ESM.pdf]
